# The evolution of functional complexity within the β-amylase gene family in land plants

**DOI:** 10.1186/s12862-019-1395-2

**Published:** 2019-02-28

**Authors:** Matthias Thalmann, Mario Coiro, Tiago Meier, Thomas Wicker, Samuel C. Zeeman, Diana Santelia

**Affiliations:** 1 Department of Plant and Microbial Biology, University of Zürich, Zollikerstrasse 107, CH-8008 Zürich, Switzerland; 2 Department of Systematic and Evolutionary Botany, University of Zürich, Zollikerstrasse 107, CH-8008 Zürich, Switzerland; 3 Department of Biology, ETH Zürich, Universitätstr. 2, CH-8092 Zurich, Switzerland; 4 Present address: John Innes Centre, Norwich Research Park, Norwich, NR4 7UH UK; 5 Present address: ETH Zürich, Institute of Integrative Biology, Universitätstrasse 16, 8092 Zürich, Switzerland

**Keywords:** Green plants, Phylogenetic analysis, β-Amylase, Gene duplication, Functional diversification, Starch

## Abstract

**Background:**

β-Amylases (BAMs) are a multigene family of glucan hydrolytic enzymes playing a key role not only for plant biology but also for many industrial applications, such as the malting process in the brewing and distilling industries. BAMs have been extensively studied in *Arabidopsis thaliana* where they show a surprising level of complexity in terms of specialization within the different isoforms as well as regulatory functions played by at least three catalytically inactive members. Despite the importance of BAMs and the fact that multiple BAM proteins are also present in other angiosperms, little is known about their phylogenetic history or functional relationship.

**Results:**

Here, we examined 961 β-amylase sequences from 136 different algae and land plant species, including 66 sequenced genomes and many transcriptomes. The extraordinary number and the diversity of organisms examined allowed us to reconstruct the main patterns of β-amylase evolution in land plants. We identified eight distinct clades in angiosperms, which results from extensive gene duplications and sub- or neo-functionalization. We discovered a novel clade of BAM, absent in Arabidopsis, which we called BAM10. BAM10 emerged before the radiation of seed plants and has the feature of an inactive enzyme. Furthermore, we report that BAM4 – an important protein regulating Arabidopsis starch metabolism – is absent in many relevant starch-accumulating crop species, suggesting that starch degradation may be differently regulated between species.

**Conclusions:**

BAM proteins originated sometime more than 400 million years ago and expanded together with the differentiation of plants into organisms of increasing complexity. Our phylogenetic analyses provide essential insights for future functional studies of this important class of storage glucan hydrolases and regulatory proteins.

**Electronic supplementary material:**

The online version of this article (10.1186/s12862-019-1395-2) contains supplementary material, which is available to authorized users.

## Background

β-Amylases (EC 3.2.1.2) are hydrolytic enzymes that cleave α-1,4 glucosidic bonds at the non-reducing end of polyglucan chains to produce maltose. β-Amylases are found in eukaryotes and bacteria. Amongst eukaryotes, they are absent in fungi and animals (Ophistokonta) but present in most other clades, including plants (Archaeplastida). Previous research has shown that plant β-amylases (BAM) originated from the eukaryotic host and not, as the case for many plant genes, from the cyanobacterial endosymbiont which gave rise to the plastid [1]. Plant genomes encode multiple β-amylase-like proteins, but not all are active enzymes. Several catalytically inactive paralogs, so called pseudoenzymes, have been identified [2], including two transcription factors [3]. Attempts to reconstruct the phylogeny of plant β-amylases have resulted in conflicting topologies. Some studies identified four major subfamilies, according to sequence similarities, gene structure and the conservation of the intron positions [2, 4]. More recently, studies based on the intron position alone found only two different subfamilies [5, 6]. Furthermore, the exact pattern of BAM gene duplication (sub- and neo-functionalization), gene loss and conservation in plants is still unclear.

The three-dimensional structure of β-amylase has been determined for soybean (*Glicine max*) [7, 8], barley (*Hordeum vulgare*) [9], sweet potato (*Ipomoea batatas*) [10], and *Bacillus cereus* [11, 12]. In all cases, β-amylase exhibits a well conserved (β/α)_8_-barrel fold in the core domain and an active site in the cleft of the barrel. The enzymatic hydrolysis of the glucosidic bond is a general acid-base catalysis involving two glutamic acid (Glu) residues. In the soybean enzyme, Glu-186 acts as a general acid, while Glu-380 acts as a general base [8, 13]. Structural analysis of the soybean β-amylase-maltose complex indicated that the carboxyl group of Glu-186 is located on the hydrophilic surface of the glucose and protonates the glucosidic oxygen [7]. Subsequently, the deprotonated Glu-186 is stabilized by threonine-342 (Thr-342) located in the inner loop [13]. The carboxyl group of Glu-380 lies on the hydrophobic face of the glucose residue at the subsite − 1 and activates the attacking water molecule, which ultimately leads to the cleavage of the glycosidic bond [7, 8]. In the case of *B. cereus*, Glu-172 and Glu-367 act as the general acid and base catalyst, respectively, corresponding to Glu-186 and Glu-380 in soybean β-amylase [11]. In addition to these regions directly involved in the catalytic reactions, a fourth region – the flexible loop – corresponding to amino acids 96–103 of the soybean enzyme, is essential for binding of the glucan chain and enzymatic activity [7, 8]. The reducing glucose of the released maltose is in the β-form, explaining the name β-amylase.

Most studies investigating the function of β-amylases in vivo have been conducted in the model plant *Arabidopsis thaliana*. The Arabidopsis genome contains nine BAM isoforms (Table 1). At least four of them (*At*BAM1 to *At*BAM4) are targeted to the chloroplast [2, 14]; two more (*At*BAM7 and *At*BAM8) are nuclear proteins [3], while *At*BAM5 is a cytosolic protein and is mainly found in the sieve elements in the phloem [15, 16]. The subcellular localization and the physiological function of *At*BAM6 and *At*BAM9 are so far unknown.

**Table 1:** The Arabidopsis β-amylase gene family

Gene	AGI code	Amino acids	Active enzyme	Demonstrated localization	References
***BAM1***	*At3g23920*	575	Yes	Chloroplast	(Kaplan and Guy 2005; Sparla et al. 2006; Valerio et al. 2011; Monroe et al. 2014; Prasch et al. 2015; Horrer et al. 2016; Zanella et al. 2016; Thalmann et al. 2016)
***BAM2***	*At4g00490*	553	K ^+^ -dependent	Chloroplast	(Fulton et al. 2008; Monroe et al. 2017)
***BAM3***	*At4g17090*	548	Yes	Chloroplast	(Kaplan and Guy 2005; Fulton et al. 2008; Monroe et al. 2014; Horrer et al. 2016; Zanella et al. 2016; Thalmann et al. 2016; Li et al. 2017)
***BAM4***	*At5g55700*	531	No	Chloroplast	(Fulton et al. 2008; Li et al. 2009)
***BAM5***	*At4g15210*	498	Yes	Cytosol	(Wang 1995; Laby et al. 2001)
***BAM6***	*At2g32290*	577	–	–	–
***BAM7***	*At2g45880*	691	No	Nucleus	(Reinhold et al. 2011; Soyk et al. 2014)
***BAM8***	*At5g45300*	689	No	Nucleus	(Reinhold et al. 2011; Soyk et al. 2014)
***BAM9***	*At5g18670*	536	–	–	–

Several β-amylases are key enzymes of plastidial starch degradation. This is illustrated by the *s*tarch *ex*cess (*sex*) phenotype of Arabidopsis plants lacking chloroplastic β-amylase isoforms [2, 17] as well as by the rapid accumulation of their product maltose during the night when starch is degraded [2, 18]. Of the four β-amylases known to localize to the chloroplast, *At*BAM1 and *At*BAM3 are catalytically active and their respective recombinant proteins have high specific activities on glucan substrates in vitro [2, 4, 19]. *At*BAM2 activity is greatly increased by potassium and exhibits cooperative kinetics. Without potassium or at low concentration of starch its activity is negligible [5]. Conversely, *At*BAM4 appears to be non-catalytic due to several amino acid substitutions within its active site, including one of the two catalytic glutamate residues [2].

Under standard growth conditions, mutants of *At*BAM3 show a mild *sex* phenotype, whereas mutants of *At*BAM1 have no obvious alterations in leaf starch metabolism compared to wild-type plants [2]. Additionally, *At*BAM3 has been implicated in cold stress-induced starch degradation [17], whereas *At*BAM1 is involved in starch degradation in guard cells during stomatal opening [20] and tolerance to osmotic stress and heat stress [4, 21–24]. Despite the observed sub functionalization, *At*BAM1 and *At*BAM3 have partially overlapping functions, as demonstrated by the fact that the *bam1bam3* double mutant has a more severe *sex* phenotype than the *bam3* single mutant [2]. Thus, *At*BAM3 is the major isoform during night-time starch degradation, but *At*BAM1 can also contribute to this process, at least in the absence of *At*BAM3.

Although *At*BAM4 protein has no detectable β-amylase activity, Arabidopsis *bam4* mutants show impaired starch degradation. It is unclear how a non-catalytic β-amylase-like protein could influence starch breakdown. It has been speculated that *At*BAM4 could act as a chloroplastic regulator, potentially responding to the concentration of maltose, and thereby fine-tuning the rate of starch degradation [2]. Alternatively, *At*BAM4 could mediate starch degradation by acting as a scaffold protein facilitating the binding to starch of other hydrolytic enzymes [25]. Direct evidence for either function is lacking.

*At*BAM2 is an active enzyme, but in leaves of five-week-old plants, no change in phenotype could be observed when the protein was missing either alone or in combination with other β-amylases [2]. However, eight-week-old leaves of Arabidopsis *bam2* mutants show a *sex* phenotype, indicating a specific role at this developmental stage [4].

Many β-amylase-like proteins are not involved in starch metabolism. It was shown that two of them, *At*BAM7 and *At*BAM8, are localized to the nucleus and possess an additional Brassinazole Resistant 1 (BZR1)-type DNA binding domain [3]. These proteins act as transcriptional regulators affecting shoot growth and development by interacting with brassinosteroid signaling, but have no direct influence on starch degradation. It was suggested that the β-amylase-like domain could act as a metabolite sensing domain rather than catalyzing the hydrolysis of glucans like true β-amylases [3]. Further evidence for this model was provided by Soyk et al. [26], who showed that eradicating the residual enzymatic activity by the substitution of Glu-429 of *At*BAM8 in Arabidopsis (corresponding to Glu-180 in the soybean enzyme) led to no change in the transcription factor activity. In contrast, the amino acid substitution of Glu-623 (Glu-380 in soybean BAM), which was predicted to prevent substrate or ligand binding, caused a drastic reduction of the transcriptional activator function of *At*BAM8 [26].

The cytosolic *At*BAM5 appears not to be involved in starch breakdown either, as the corresponding *bam5* mutants have normal starch levels [16]. It was speculated that *At*BAM5 might be involved in digesting starch granules released from the plastids of the phloem sieve elements as they differentiate into open tubes [15].

In contrast to the detailed analysis performed in Arabidopsis, relatively little is known about the physiological role of β-amylases in most other plants, including commercially relevant crop species. The existing data indicate that they play an important role in plastidial leaf starch turnover in rice (*Oryza sativa*) [27] and potato (*Solanum tuberosum*) [28]. In the rice genome, there are nine genes predicted to encode β-amylase-like proteins [29, 30]. Of these genes, at least *OsBAM2* (*Os10g0465700*) and *OsBAM3* (*Os03g0141200*), which are closely related to *At*BAM1, encode plastid-targeted active isoforms [30]. The overexpression of these isoforms leads to reduced starch accumulation in the third leaf sheaths at the heading stage and stunted plant length [27]. However, knockdown of the individual genes did not result in excess accumulation of starch in the leaf sheaths, suggesting redundancy between these two isoforms or the presence of a complementary function of another gene encoding a starch-degrading enzyme [27].

In cereal seeds, β-amylases have been studied because of their economic importance in the brewing industry. They are the major factor in determining the malting quality of the grain. Their activity is essential for the generation of maltose and other easily fermentable sugars from cereal grain starch in the mashing process to fuel the production of alcohol by yeast [31]. Despite such agronomic interest, the genetics of cereal seeds β-amylase has been insufficiently studied to date, impeded by the gene redundancy associated with the complexity and polyploidy of the genomes of cereal species. Thus, the exact physiological function of cereal seeds β-amylase is still not understood and most of our current knowledge derives from early biochemical work. It was shown that β-amylase accumulates in the cytosol of the endosperm cells in both “free” and “bound” forms [31]. During seed germination, “bound” β-amylase is released in a soluble active form by limited proteolysis or disulphide reduction, resulting in a transient increase in total β-amylase activity [32, 33]. However, soybean, rye (*Secale cereale*) and barley mutants that lack active β-amylase or contain only traces of activity germinate normally [34–36].

These studies reveal a surprising level of complexity of plant β-amylase function, supporting the hypothesis that BAM proteins have diversified during the course of land plant evolution. Here, we investigated the origin of plant β-amylases and their pattern of genes duplication, loss and conservation amongst the different lineages of land plants. We computationally identified 961 BAM ortholog sequences from 136 different species, including algae and land plants, in a mixture of both genomic and transcriptomic data, and reconstructed the evolutionary history of the β-amylase gene family. Our work reveals the molecular basis of the functional divergence of BAM genes from different lineages of seed plants, providing an essential platform for future molecular evolution and functional studies of this important class of storage glucan hydrolytic and regulatory enzymes.

## Methods

### Identification of β-amylase ortholog sequences

Conserved β-amylase protein sequences were identified using BLAST [37] blastp algorithm in default parameter settings, with Arabidopsis BAM1 as query sequence. Multiple databases were screened, including the National Center for Biotechnology Information (NCBI, https://www.ncbi.nlm.nih.gov/), Phytozome v10.3 (http://phytozome.jgi.doe.gov/pz/portal.html), and the websites of authors (listed in Additional file 1: Table S1). Additionally, β-amylase sequences from acrogymnosperms, ferns, basal embryophytes and the green algae charophytes were retrieved from the transcriptomes available in the 1000 Plants project (OneKP) database (https://sites.google.com/a/ualberta.ca/onekp/home; for details see Additional file 1: Table S1, Additional file 2). BAM-like proteins were identified as having significant E-values (usually less than 10^− 100^) and preserving the known conserved catalytic domain (according to UniProt, http://www.uniprot.org/uniprot/Q9LIR6). The identified sequences were further examined manually to eliminate spurious hits, and a total of 961 BAM proteins from 136 archeaplastida species were used for comparative and evolutionary analyses. The retrieved BAM sequences were aligned with BAM sequences from *A. thaliana*, *Amborella trichopoda* and *Solanum lycopersicon*, and a preliminary approximate maximum-likelihood tree was used to manually distinguish identified homologs. If multiple sequences from a single species showed no amino acid polymorphisms but lengthy insertions/deletions or differed in their start codon, they were assumed to represent different potential gene models. In such cases, the most parsimonious model was used for further analysis and the other sequences were discarded. Short sequences (less than 100 amino acids) were also excluded from further analysis.

### Multiple sequence alignment

The full-protein sequences of the retrieved BAMs were aligned using MAFFT [38] with default settings. The derived alignment was then subject to visual inspection and manual editing in Molecular Evolutionary Genetics Analysis (MEGA) 6.0 program [39].

Three different matrices were generated. The first (Matrix A) included most sequences from land plants (Embryophyta), and was designed to test the relationships between the major BAM classes and their history of losses and duplications. We decided to remove from the alignment the *N*-terminal part of the sequences up to the position corresponding to G105 of *At*BAM1, since this region was extremely variable in length and amino acid composition and difficult to align.

The second matrix (Matrix B) included a more thorough sampling of sequences from the Bryophyta, as well as seed plant sequences of BAM classes which were not found in the bryophyte genomes. The following representative seed plant species were selected, as they are well spread across the different seed plant lineages and their genome is well annotated: Arabidopsis (*A. thaliana*), poplar (*Populus trichocarpa*), tomato (*Solanum lycopersicum*), date palm (*Phoenix dactylifera*), *Brachypodium distachyon*, *Amborella trichopoda* [40–44]. We also included sequences from the Rhodophyta, Chlorophyta and Charophyta.

Finally, to identify the origin of the BZR domain in BAM8 and BAM7, an additional matrix was generated (Matrix C) that included BZR-domain proteins from a subset of land plants, as well as some of the BZR-BAM proteins (See Additional file 2). This alignment was subjected to cleaning using the Gblocks server [45], as the regions outside the BZR domain are not homologous between all sequences and cannot be aligned in a meaningful way.

All matrices and phylogenetic trees are available on figshare (https://figshare.com/s/87b2fcd1813587d6bb41).

### Phylogenetic analyses and detection of amino acid polymorphism sites and conserved sites

ML trees were generated using PhyML [46], IQTREE [47], and RAxML [48]. PhyML was run on the PhyML web browser [46], whereas RAxML and IQTREE were run on the CIPRES Cyberinfrastructure [49]. Model selection for the PhyML runs was conducted using the SMS method [50] and using AIC scores. For the IQTREE runs model selection was conducted using ModelFinder [51] as implemented in the CIPRES implementation of the software. RAxML was run using the best model found in the PhyML model selection. Support for the nodes was established by fast bootstrapping using 500 replicates for the RAxML runs [52], Ultrafast bootstrap with 1000 replicates for the IQTREE runs [53] and approximate likelihood-ratio test using the Shimodaira-Hasegawa-like estimate (SH-like aLRT) for PhyML [54]. Support in the text is shown as RAxML fast bootstrap/IQTREE Ultrafast bootstrap/PhyML SH-like aLRT, unless otherwise stated.

The amino acid polymorphism sites and conserved sites were analyzed by WebLogo (http://weblogo.berkeley.edu/logo.cgi) [55], through which sequence logos were generated according to alignment.

### Prediction of subcellular localization

The presence of possible chloroplast transit peptides was predicted using ChloroP1.1, a neural network–based method for identifying targeting information in peptide sequences [56]. Nuclear localization signals were predicted using NLStramadus [57]. As the transit peptide is always at the very *N*-terminus of a protein and the nuclear localization signal in Arabidopsis BAM7 and BAM8 is likewise found in the *N*-terminal part of the protein [3], only protein sequences covering the full *N*-terminal region were included in this analysis.

## Results

### Identification and distribution of β-amylase family members across algae and land plants

To investigate the origin and the evolutionary history of the plant β-amylase gene family, we retrieved the available BAM-like protein sequences from currently sequenced and unfinished genomes as well as transcriptomic databases, using the Arabidopsis BAM1 as a query sequence. 961 BAM-like ortholog sequences were identified from 136 different species representing algal and land plant lineages (see Additional file 1: Table S1 and Additional file 2). All species queried contained multiple copies of BAM-like sequences, with copy number being lower in algal species and higher in land plants, with the highest copy numbers found into flowering plant species (Additional file 2 and Additional file 3). For most species, the predicted BAM-like protein sequences ranged from approximately 500 to 700 amino acids, beginning with an initiation codon and ending with a stop codon. However, for some species, deletions or truncations were observed, mostly because the sequences were derived from fragmented transcriptome assemblies.

### Eight distinct BAM clades were already present in the ancestor of flowering plants

**Fig. 1:**
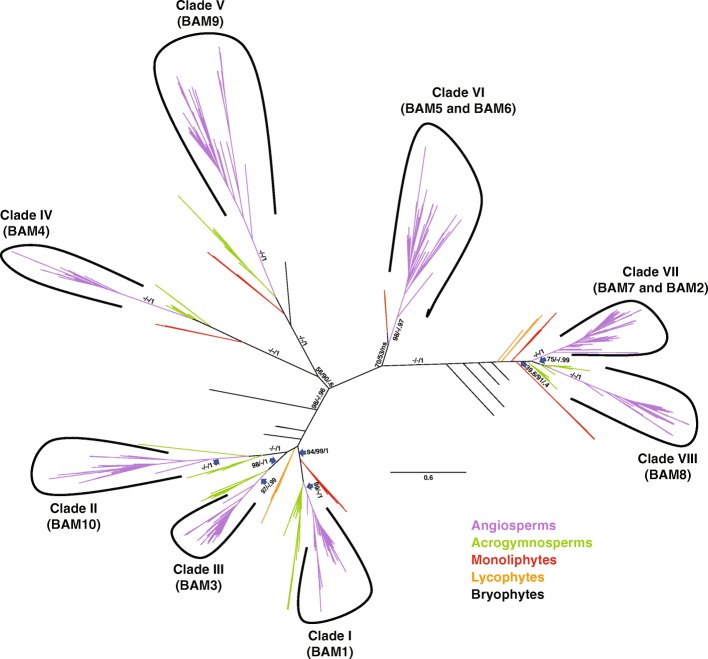
Phylogeny and classification of β-amylases in land plants. The Maximum-Likelihood tree from the IQTREE analysis of the trimmed matrix of 834 BAM proteins from 115 representative land plant species is shown. The information of species and sequences accession numbers used for the tree are listed in Additional files 1 and 2. BAMs from angiosperm are clustered into eight well supported clades, which are identified by Latin numbers (I to VIII). Support values (RaxML fast bootstrap/IQTREE ultrafast bootstrap/ PhyML SH-like aLRT) are shown over relevant branches. The scale bar represents amino acid substitutions per site

We used Matrix A including most sequences from land plants (Embryophyta) to test the relationship between the major BAM classes and their history of losses and duplications. The model selection analysis for Matrix A retrieved similar models with both approaches (JTT + G + I using SMS and JTT + R9 using ModelSelect). The trees obtained from the phylogenetic analyses allowed us to subdivide the previously identified four plant β-amylase subfamilies [2, 4] into eight distinct clades (Fig. 1). Clade I included *At*BAM1 and its orthologs, and it was strongly supported in all analyses (99/100/1). Clade II was composed of a previously unidentified β-amylase isoform, surprisingly absent in Arabidopsis, which we named BAM10 (Fig. 1); the clade of BAM10 orthologs from angiosperms was strongly supported in all analyses (100/100/1). Clade III consisted of *At*BAM3 and its orthologs, and it was strongly supported in all analyses (97/100/0.99). The branch separating these three clades from the rest of the BAMs received strong bootstrap support (98/100/0.95, Fig. 1). Despite the orthologs of *At*BAM4 and *At*BAM9 clustered in a closely related branch, such branch was only strongly supported in the IQTREE analysis (56/90/0.6). Thus, we conclude that they form two individual clades that we called clade IV (BAM4) and V (BAM9; Fig. 1), a conclusion which is also supported by the different intron-exon positions [5]. These two clades were both strongly supported (100/100/1). Clade VI contained both *At*BAM5 and *At*BAM6 as well as their orthologs (Fig. 1). While the clade of angiosperm-specific BAM5 sequences was strongly supported (98/100/0.97), the grouping of the few acrogymnosperm and monilophyte BAM5-like genes only received support in the RAxML bootstrap analysis (70/53/ns). Clade VII contained *At*BAM2 and *At*BAM7 together with their orthologs, while clade VIII contained *At*BAM8 and its orthologs. Clade VII (BAM2, BAM7) appears to be angiosperm-specific, since all acrogymnosperm sequences either clustered with clade VIII (75.5/100/0.99) or were in a clade sister to clade VII plus clade VIII (39.6/91/0.4; Fig. 1). An intriguing feature of the two clades is that the placement of genes is independent of the presence of a DNA-binding domain (BZR-domain). Sequences of flowering plants all fell within Clade VII or VIII, regardless of whether they contain a BZR-domain (e.g. BAM7 and BAM) or not (e.g. BAM2). Likewise, sequences of gymnosperms and monilophytes clustered in separate clades regardless of whether they contained a BZR-domain or not.

Representative BAM sequences of each of these eight clades were found in the genome of most sequenced angiosperms (Fig. 1, violet branches; Additional file 2 and Additional file 3), indicating that eight distinct β-amylase clades were present already in the ancestor of flowering plants. In contrast, BAMs from the analyzed acrogymnosperms showed representative sequences for some, but not all clades (Fig. 1, green branches; Additional file 2 and Additional file 3). The eight plant BAM clades are thought therefore to have emerged before the radiation angiosperms, and subsequently conserved in most of the extant representative angiosperms.

### The different *BAM clades emerged during the evolution of land plants but had not yet diverged in algae*

The model selection analysis of matrix B favored a different replacement matrix compared to matrix A (LG + G + I using SMS and LG + R9 using ModelSelect).

**Fig. 2:**
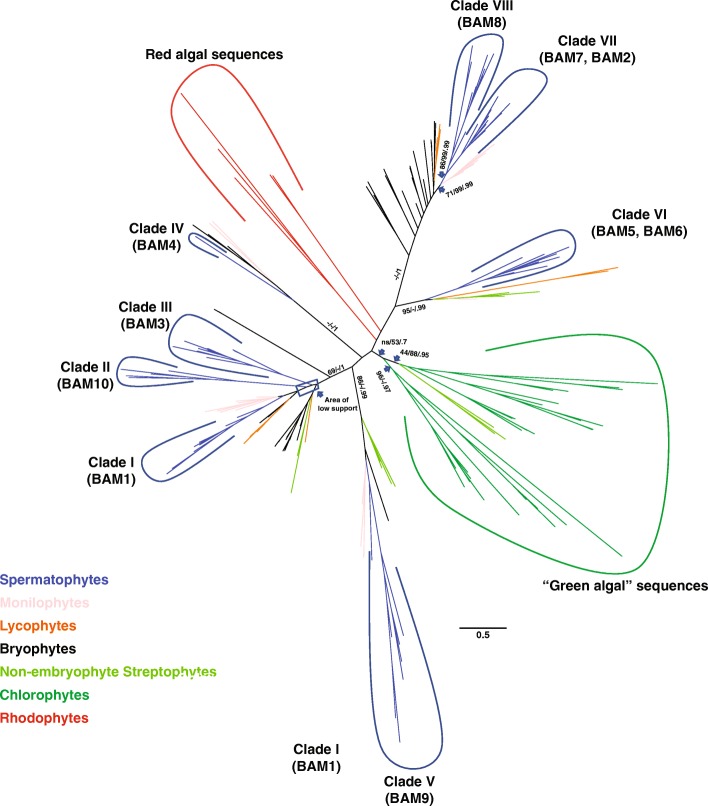
Evolutionary origin of the eight plant β-amylase clades. The unrooted Maximum-Likelihood phylogenetic tree from IQTREE of the trimmed matrix of 160 BAM proteins from 40 species, including algae, lower land plants and representative seed plants is shown. Detailed information of species and sequences accession numbers used for the tree are listed in Additional files 1 and 2. Only relevant support values are shown. The scale bar represents amino acid substitutions per site

As shown by the contrasting topologies in Figs. 1 and 2, the relationship between clades I-III (BAM1, BAM3 and BAM10) are difficult to establish, probably due to low signal in the data. Sequences from bryophytes, lycophytes, monilophtes as well as non-embryophyte streptophytes were all in a clade with the three angiosperm clades and their acrogymnosperm orthologs (support 69/100/1). However, the three angiosperm clades did not cluster together, as sequences from more basal species are interspersed between them.

Orthologs of clade IV (BAM4) were identified also in the transcriptome of hornworts (e.g. *Nothoceros spp.*), liverworts (e.g. *Marchantia spp.* and *Treubia lacunosa*) and monilophytes, while orthologs of clade V and VI (BAM5, BAM6 and BAM9) were found in the transcriptome of other lycophytes (e.g. *Huperzia spp.*) and in liverworts (Fig. 2). Based on this phylogeny, we suggest that plant BAM clades I-III must have diverged more recently compared to BAM clades IV, V and VI.

Clades VII and VIII appear to have diverged after the appearance of the spermatophytes, since the angiosperm sequences from this clade plus the one acrogymnosperm sequence for this clade present in matrix B formed two sister clades (86/99/0.99). Most of the sequences from bryophytes were placed as successive sisters to a split including a clade of monilophyte sequences as sister to clade VII and VIII (71/99/0.99); Fig. 2). Taken together, our results place the emergence of clade IV, clade V, and the ancestral β-amylase domain giving rise to BZR-BAMs before the radiation of land plants, while the origin of clades I, II and III potentially postdated the origin of vascular plants.

The precise origin of clades I, II and III is however made unclear by the low support of the relationships between them, with different sampling strategies (matrix A vs matrix B) and different methods giving different, non-compatible answers. This lack of signal could be a consequence of strong functional divergence between the members of the three clades. An origin of clades I, II and III before the evolution of seed plants would have to imply extensive loss of genes more closely related to clades II and III in bryophytes, lycophytes and monilophytes, which is unlikely. Thus, an origin postdating the spermatophytes could represent the most parsimonious option.

The green algae most closely related to land plants (non-embryophyte streptophytes) contained unique algal BAM-like sequences which shared only little similarity with BAMs from land plants. However, one non-embryophyte streptophyte clade was nested in the clade comprising BAM1, BAM3 and BAM10. The clade grouping this clade with BAM1, BAM3 and BAM10 and sequences from basal land plants was well-supported (69/−/1), suggesting that the ancestral gene that gave rise to these three spermatophyte forms already existed before the origin of land plants (Figs. 1 and 2). Likewise, another clade containing non-embryophyte streptophyte BAM-like sequences was more related to clade VI (Fig. 2). β-amylases from the chlorophytes clustered in two clades, one including only sequences from chlorophytes (44/88/0.95) and the other including also sequences from streptophytes (95/100/0.97). These two clades were sister in all ML trees, but this relationship received weak to no support (ns/53/0.77). Sequences from rhodopyhtes were even more divergent. However, a clade of rhodophyte sequences was not supported by the data.

### The enzymatic activity but not the subcellular localization is conserved within each plant β-amylase clade

The amino acid residues and the specific protein sites harboring short amino acid motifs that are important for β-amylase catalytic activity have been previously identified and demonstrated to be strictly conserved amongst active plant BAM enzymes [7–10]. To investigate the degree of conservation of these residues amongst plant BAM orthologs within each clade, we assessed their sequence characteristics (i.e. the amino acid polymorphism) using WebLogo [55].

**Fig. 3:**
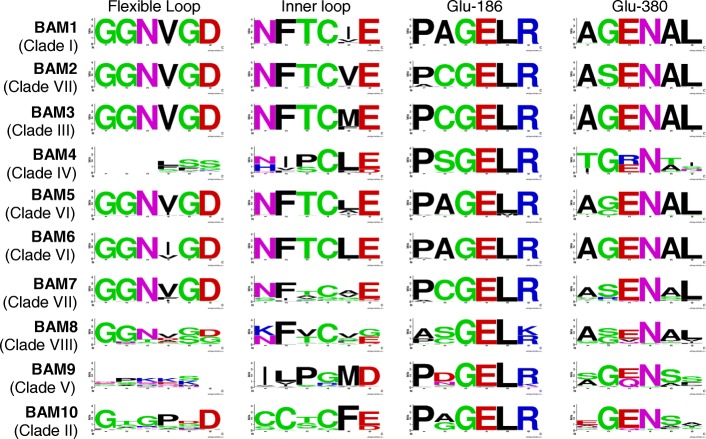
Architecture of conserved protein motifs in the ten isoforms of the plant β-amylase gene family. The sequence logos of the amino acid motifs important for BAM catalytic activity within the flexible loop, inner loop and surrounding Glu-186 and Glu-380 are shown. The flexible loop covers amino acids 340–346, while the inner loop amino acids 96–103. The clades to which each BAM isoform belongs to are indicated in parenthesis. The bit score indicates the information content for each position in the sequence. The height of the letter designating the amino acid residue at each position represents the degree of conservation. Sequence logos were created using WebLogo (Crooks et al., 2004)

Orthologs of the catalytically active *At*BAM1 and *At*BAM3 (clades I and III, respectively) showed highly conserved amino acid motifs for all the regions known to be involved in catalysis (i.e. the flexible loop and inner loop, and the Glu residues corresponding to Glu-186 and Glu-380 in soybean β-amylase [7, 8] (Fig. 3)), suggesting that clades I and III contain active β-amylases. Orthologs of *At*BAM2, forming a subset of Clade VII, likewise showed conserved motifs in these regions, in line with recent reports that *At*BAM2 is an active enzyme [5]. Conversely, the sequence logos of BAM ortholog proteins belonging to clades IV (BAM4), clade VII (BAM7) and clade VIII (BAM8) contained many amino acid substitutions and had very low bit scores, indicating a poor degree of conservation (Fig. 3). In particular, the inner loop, the catalytic residue Glu-380 and its surroundings amino acids were poorly conserved in all three clades (Fig. 3). These results are consistent with the fact that the corresponding Arabidopsis ortholog proteins, *At*BAM4, *At*BAM7 and *At*BAM8, are catalytically inactive [2, 3]. However, we noticed that the second catalytic residue (Glu-186) was conserved even in catalytically inactive BAM proteins (Fig. 3), suggesting that it might be required not just for catalysis but also for other functions. Moreover, while the flexible loop was heavily substituted in *At*BAM4 orthologs from clade IV, this was still largely conserved in BAM sequences from clades VII and VIII (Fig. 3). Taken together, our findings are in line with the demonstrated activity of the respective Arabidopsis isoforms, and indicate that plant BAM orthologs belonging to the same clade are generally highly conserved in terms of catalytic function.

We also analyzed the catalytic residues in *At*BAM6 and *At*BAM9 and their orthologs (belonging to clade VI and V, respectively), for which no information is available regarding their biochemical or enzymatic properties. Our analysis revealed that all residues important for catalysis were conserved in *At*BAM6 orthologs, while orthologs of *At*BAM9 showed numerous mutations in key residues (Fig. 3). Based on these findings, we speculate that clade VI contains catalytically active BAMs, while BAMs orthologs from clade V are presumably inactive proteins.

Next, we investigated the predicted sub-cellular localization. *In-silico* analysis indicated that most BAM isoforms from clade I, IV, and VIII localize to the same compartment as their Arabidopsis orthologs (Table 2, Additional file 4: Tables S2, S5 and S9). Clade VII contains two Arabidopsis orthologs, *At*BAM2 and *At*BAM7 (Fig. 1). *At*BAM2 has been shown to be a plastidial protein [2], while *At*BAM7 is a nuclear protein [3]. According to our prediction, this sub-cellular localization is retained by most of their respective BAM orthologs, i.e. *At*BAM2 orthologs localize to the plastid and *At*BAM7 orthologs to the nucleus (Table 2, Additional file 4: Table S3 and S8). The same holds true for *At*BAM5 orthologs from Clade VI, most of which are predicted to be cytosolic proteins similar to Arabidopsis isoform (Table 2, Additional file 4: Table S6) [16]. However, orthologs of *At*BAM6 from the same clade, which form a Brassicacea-specific subclade, are predicted to be plastidial proteins (Table 2, Additional file 4: Table S7). Thus, our in silico-analysis suggests that BAM isoforms belonging to the same clade can localize to a different sub-cellular compartment, but the localization of individual isoforms matches in each case that of the corresponding Arabidopsis orthologs. An exception is *At*BAM6 from clade VI, for which there is no information about its in vivo sub-cellular localization.

**Table 2:** Predicted localization of β-amylases from different clades

Clade	Arabidopsis ortholog	Predicted localization
I	BAM1	Plastidial (68/92)
II	–	Plastidial (40/40)
III	BAM3	Plastidial (45/82)
IV	BAM4	Plastidial (22/30)
V	BAM9	Cytosolic (44/77)
VI	BAM5	Cytosolic (63/77)
	BAM6	Plastidial (7/11)
VII	BAM2	Plastidial (35/39)
	BAM7	Nuclear (42/49)
VIII	BAM8	Nuclear (49/49)

**Note.** In parenthesis is indicated the number of sequences matching the predicted localization out of the total number of analyzed sequences

Unfortunately, no conclusive predictions could be obtained for clades III (BAM3) and V (BAM9). While *At*BAM3 is a plastidial protein [2], only 55% of its ortholog proteins were predicted to share this localization (Table 2 and Additional file 4: Table S4). The localization of *At*BAM9 has not been experimentally verified so far. However, only 45% of the *At*BAM9 ortholog sequences queried were predicted to contain a transit peptide, whereas the remainder were predicted to be cytosolic (Table 2 and Additional file 4: Table S10). It is unclear whether these inconsistencies are due to artefacts generated by the bioinformatics analysis or if they reflect a genuine difference in subcellular localization between different orthologs from the same clade.

### *A new plant BAM clade was identified, which is absent in* Arabidopsis

**Fig. 4:**
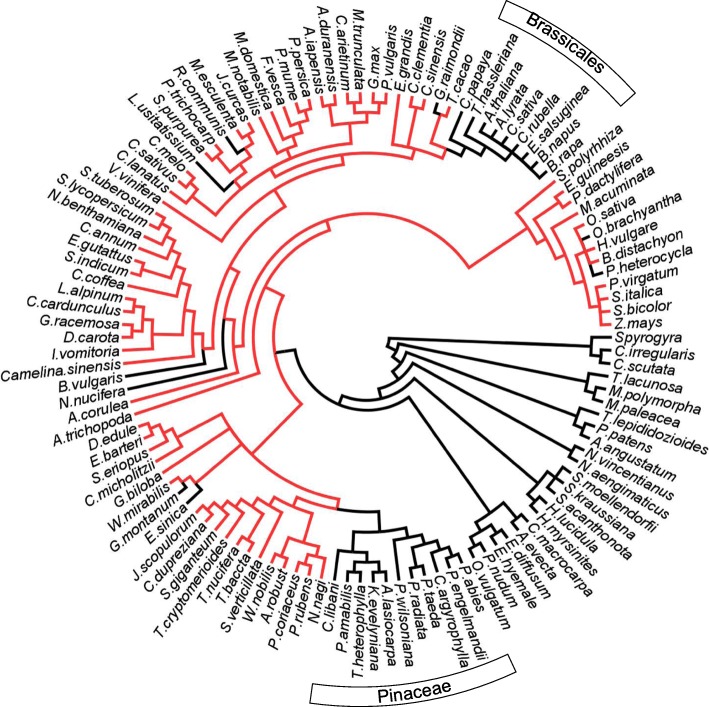
Emergence and loss of the newly discovered BAM10 across the evolution of land plants. The species relationships were redrawn according to (Ruhfel et al., 2014). Branches including species in which BAM10 has been identified are highlighted in red, while black branches refer to species in which BAM10 was not found. BAM10 is present in almost all spermatophytes, but is absent in Pinaceae and Brassicales

Our phylogenetic analysis revealed the presence of a novel clade of plant β-amylase (here named clade II), containing isoforms which we named BAM10 (Fig. 1). BAM10 was not found in Brassicales (including the model plant *A. thaliana*), although BAM10 orthologs were present in most other Angiosperms (Fig. 4, Additional file 2 and Additional file 3). Amongst acrogymnosperms, BAM10 was notably absent in Pinaceae, although BAM10-like sequences were retrieved from the transcriptomes of members of the Cupressophyta [58]. Partial BAM10 sequences were also identified in *Ginkgo biloba*, *Welwitschia mirabilis*, as well as in cycads, indicating that BAM10 emerged before the radiation of seed plants (Fig. 4). Analysis of publicly accessible transcriptome data indicated that BAM10 isoform from tomato (*Solyc08g082810*) is expressed in most plant tissues (Additional file 5). Moreover, all BAM10 orthologs were predicted to localize to the plastid (Table 2 and Additional file 4: Table S11).

Alignment of BAM10 sequences with catalytically active (BAM1 and BAM3) and inactive (BAM4) β-amylases showed that BAM10 protein carries numerous amino acid substitutions within the recognized catalytic motifs, which would likely result in a catalytically inactive protein (Fig. 3, Additional file 6). In particular, the flexible loop region of β-amylases, which is known to be crucial for the formation of the substrate tunnel and binding of glucan [8], was conserved in BAM1 and BAM3 orthologs as GGNVGD but heavily substituted in BAM10 proteins (Fig. 3). Likewise, the inner loop was poorly conserved, with Thr-342 substituted with serine in many cases (Fig. 3). Thr-342 normally interacts with the catalytic Glu-186 and the glucan substrate, and its substitution to serine results in a 360-fold reduction of k_cat_ in soybean BAM1 [13]. Furthermore, while the catalytic residue Glu-380 was conserved in the majority of BAM10 proteins, the surrounding amino acids were poorly conserved (Fig. 3). In all these regions important for catalysis, BAM10 resembled more the catalytically inactive BAM4 than the active BAM1 and BAM3.

### BAM4 is absent in many species, including economically important staple crops

**Fig. 5:**
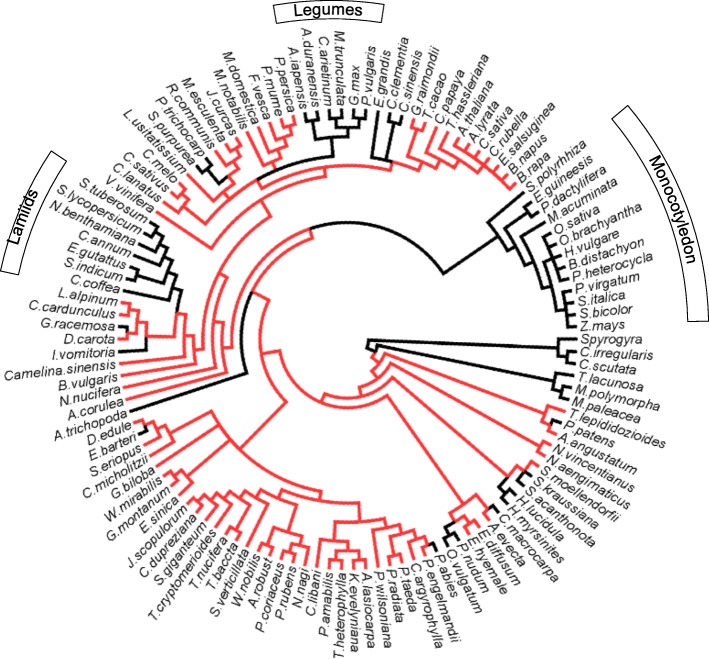
Emergence and loss of BAM4 across the evolution of land plants. The species relationships were redrawn according to (Ruhfel et al., 2014). Branches including species in which BAM4 has been identified are highlighted in red, while black branches refer to species in which BAM4 is missing. BAM4 was not found in many relevant starch-containing crop species

Despite being catalytically inactive, BAM4 plays an important regulatory role in leaf starch degradation, at least in Arabidopsis. Mutants lacking BAM4 have a starch excess phenotype [2]. Our phylogenetic analysis revealed that orthologs of *At*BAM4 are not found in any monocotyledon species, and are likewise absent in Fabaceae and Lamiids (Fig. 5, Additional file 2). Many economically important plants belong to these taxa, including all major starch-containing crops with the exception of cassava (*Manihot esculenta*). In addition to these three large families, BAM4 was also not found in Salicaceae, *Citrus* and *Eucalyptus* (Fig. 5 and Additional file 2), indicating that it might have been lost many times during the evolution of the angiosperms. Given that *At*BAM4 is essential for normal Arabidopsis leaf starch breakdown, it is surprising that BAM4 is so poorly represented in other plants.

### BZR1-BAMs likely originated in early land plants through the fusion of a β-amylase with a novel BZR/BES-like protein

**Fig. 6:**
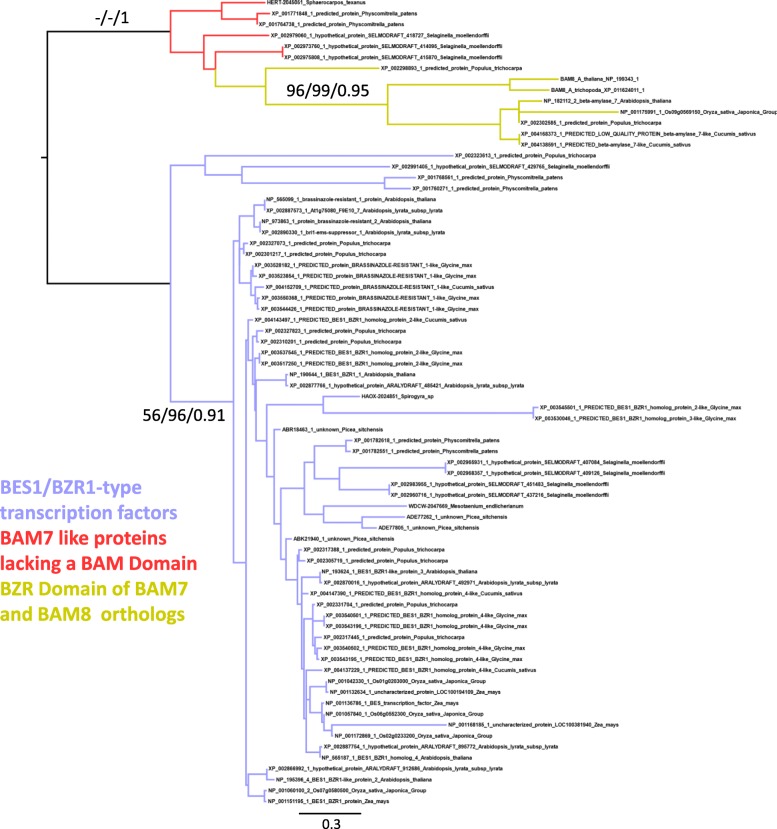
Phylogenetic relationship of BES1/BZR1 type transcription factors and the DNA-binding domain of BAM7 and BAM8. Only relevant support values are shown beside each corresponding branch. The scale bar represents amino acid substitutions per site. Support of 100 is shown as “-”, and non-supported branched (not present in the bootstrap consensus) are shown as “ns”

*At*BAM7 and *At*BAM8 are unique amongst Arabidopsis BAMs as they contain in addition to the β-amylase domain a second domain resembling the BZR1/BES1-type transcription factors, and are thus named BZR1-BAMs [3]. *At*BZR1-BAM7 and *At*BZR1-BAM8 are conserved in most flowering plants (Figs. 1, 2, 6, and Additional file 2). Furthermore, we have identified several BZR1-domain containing β-amylases in conifers, cycads as well as in the fern *Ophioglossum vulgatum* (Additional file 7). In our analysis of BES/BZR1 transcription factors and BAM7–8, (Matrix C, best models SMS JTT + G, ModelFinder JTTDCMut+G4), we identified a class of BES1/BZR1-type transcription factors in *S. moellendorffii* and *P. patens*, which was most similar to the BZR1-domain of BAM7 and BAM8. This class of novel BES1/BZR1-type genes was absent from seed plants, and clustered in a separate clade from the remaining BES1/BZR1-type transcription factors (Fig. 6). Thus, it seems that members of the Clades VII and VIII have acquired their additional BZR1 domain directly from a BZR1-type gene present in the bryophytes and lycophytes, which had already diverged from the remaining BES1/BZR1-type transcription factors before the fusion event.

### Duplications of β-amylases occurred frequently in the evolution of land plants

Since the early age of molecular evolutionary genetics [59, 60], gene duplication has been considered an important source of genetic variability in many eukaryote lineages, especially plants [61, 62]. Our findings indicate that the emergence of new β-amylase isoforms through gene duplication has been a common event during the evolution of angiosperms, significantly contributing to the expansion and functional diversification of this gene family.

Previous work suggested that BAM5 and BAM6 are the result of a recent duplication [2, 6]. Our analysis confirmed and extended this observation. BAM6 appears to have originated after a recent duplication specific for Cleomaceae plus Brassicaceae, as orthologs of BAM6 were not found outside of this family (Additional file 2). Interestingly, while BAM5 is known to be a cytosolic protein [16] and most genes in clade VI likewise lack a transit peptide, the majority of BAM6 orthologs were predicted to be localized in the chloroplast (Table 2). As the BAM6 orthologs are nested within Clade VI (Fig. 1), this suggests that the transit peptide of *At*BAM6 was acquired during or after its duplication.

**Fig. 7:**
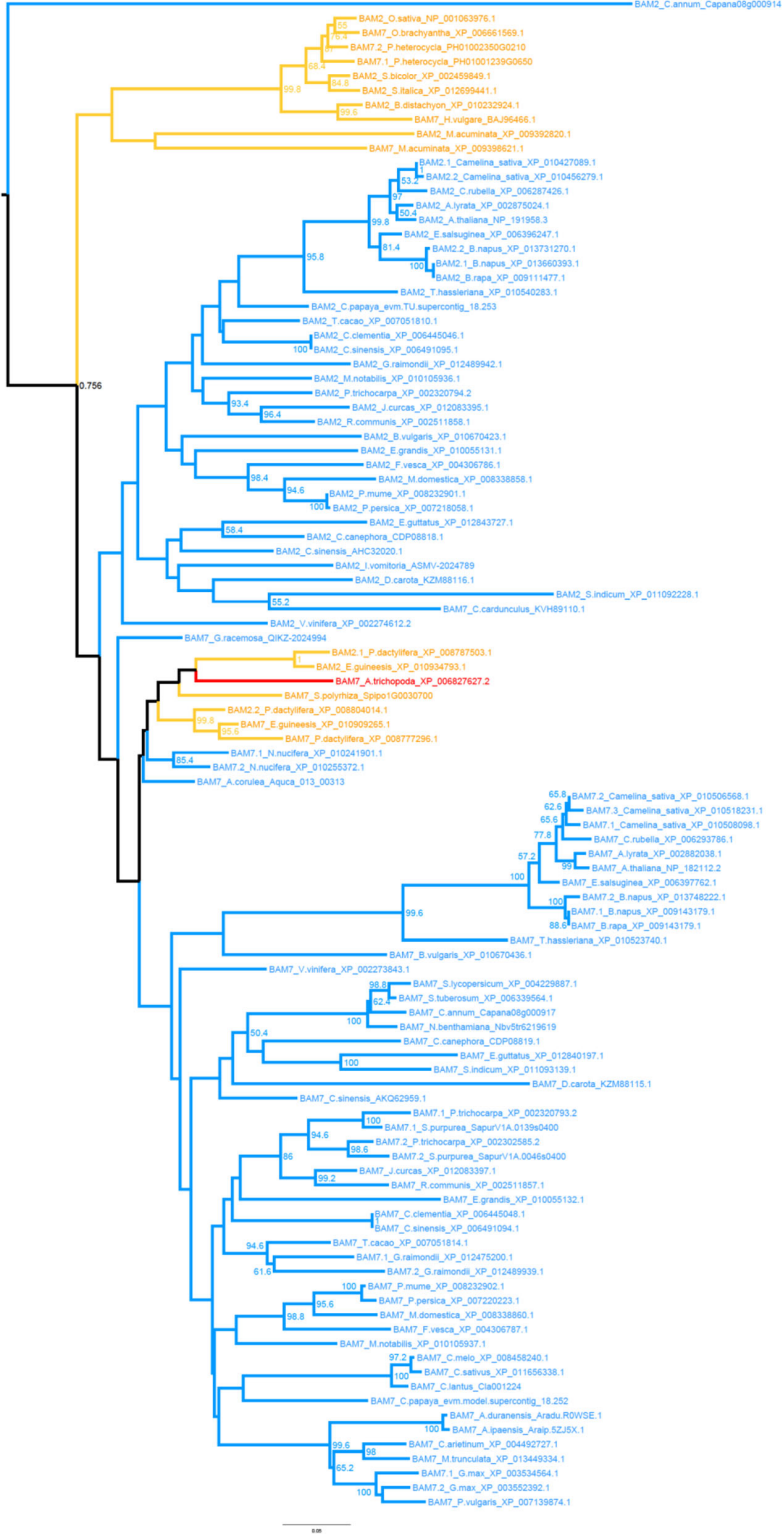
Phylogeny of β-amylase clade VII. The subtree including BAM sequences belonging to clade VII was reproduced from the phylogenetic tree of Fig. 1. Bootstrap values from 500 replications are shown beside each corresponding branch. Blue branches represent eudicotyledon sequences, yellow branches monocotyledon sequences, and the sequence of *Amborella trichopoda* is in red. The scale bar represents amino acid substitutions per site

BAM2 and BAM7 were also assumed to be the result of a recent duplication, with BAM2 being derived from BAM7 through the loss of the BZR-domain [2]. However, more recent work questioned this theory and instead proposed that BAM2 was already present in early land plants and that BAM7 is a derived form [5, 6]. Our own analysis did not recover a clade containing all BZR1-less (i.e. BAM2-like) sequences. Instead such sequences were found in different positions. Sequences form flowering plants formed clade VII together with BZR1-domain containing (i.e. BAM7-like) sequences, while BZR-less sequences from basal land plants formed their own clades subtending clade VII. Within clade VII, only the BAM2-like proteins of the Brassicaceae plus Cleomaceae form a well-supported subclade (99.6/100/1), which is likely derived from a duplication of BAM7 followed by a deletion of the BZR1-domain, as these genes were nested within BZR1-containing orthologs (Fig. 7). In contrast, BAM2-like (i.e. BZR1-domain-less) genes of grasses were more related to the BAM7 of grasses than to *At*BAM2 genes (Fig. 7). Therefore, BAM2-like genes likely represent a polyphyletic assemblage of proteins independently generated by sub-functionalization of BAM7 orthologs by loss of the BZR-like domain.

In addition to the duplications giving rise to *At*BAM2 and *At*BAM6, numerous other duplications of β-amylase genes were identified. For example, the genome of grasses encodes two paralogs of BAM1, BAM5, and BAM9 (Additional file 2). Interestingly, one of the two grasses BAM5 paralogs from Clade VI was predicted to localize to the chloroplast, like the Brassicales BAM6 (Table 2 and Additional file 4: Table S6). As the same duplication events were found in all Poaceae queried, we suggest that they most likely result from the ancestral whole-genome duplication that has been proposed for the grasses [63]. Likewise, the duplication of BAM5 found in legumes could be the result of a paleopolyploidy of the ancestral legumes [64].

Aside from the abovementioned duplications, which occurred in all species of the same family, we also identified species-specific gene duplications. In some cases, these were linked to recent polyploidization events. For example, the hexaploid *Camelina sativa* [65] contained three copies of most β-amylases, while the amphidiploid oilseed rape (*Brassica napus*) [66] contained two (Additional file 2).

## Discussion

Previous phylogenetic studies of plant β-amylases have provided valuable insights into the evolutionary history of this gene family [2, 4]. However, the limited number of sequences analyzed left many unresolved questions regarding the origin of the different BAM isoforms and their phylogenetic and functional relationship. Given the key role that β-amylases play not just for plant biology but also for many industrial applications, such as the malting process in the brewing and distilling industries [31], it is of paramount importance to disentangle the functional complexity of this gene family.

In this study, we examined 961 β-amylase sequences from 136 different algae and land plant species, including 66 sequenced genomes and many transcriptomes (Table 1 and Additional file 2). The number and the diversity of organisms examined here allowed us to identify the main patterns of β-amylase evolution in land plants. Although ongoing plant genome projects will certainly uncover additional species- or family-specific deletions and duplications, the general features are likely to not change.

**Fig. 8:**
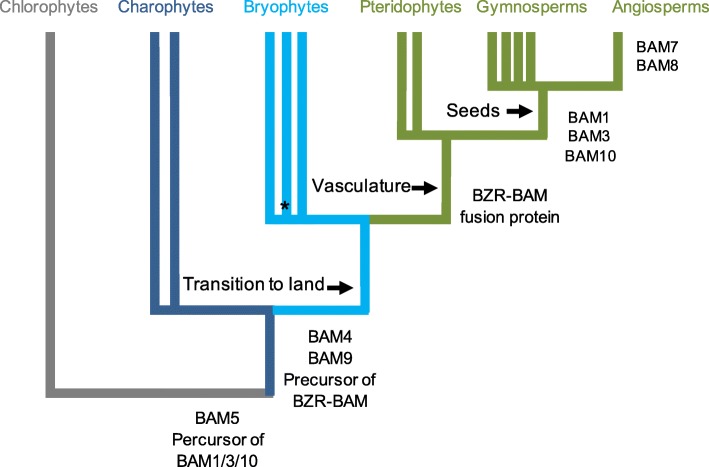
A model for the expansion and evolution of the β-amylase gene family in plants**.** Cladogram of extant land plant lineages indicating the appearance of the different BAM isoforms in relation to the evolution of key traits that marked the transition from an aquatic life to a terrestrial one. The green algae charophyte already contained BAM5 and an ancestral version of BAMs from clades I-III (BAM1/3/10-like). BAM4, BAM9 as well as at least one gene encoding for BZR-BAM were present in the ancestor of all land plants. BAM1, BAM3 and BAM10 appeared in seed plants, while BAM7 and BAM8 (the two BZR-BAMs) emerged in coincidence with the evolution of flowering plants. BAM2 and BAM6 originated from BAM7 and BAM5, respectively, from a recent duplication event. BAM6 is only present in Brassicales

Our phylogenetic analyses revealed that plant β-amylases are an extraordinary example of gene sub- and neo-functionalization of an otherwise a simple metabolic enzyme. Across all angiosperms (i.e. seed plants), we identified eight clades of β-amylases, two of which (clades VII – BAM2 and BAM7; and clade VIII – BAM8) appeared to be the result of a duplication event specific to angiosperms (Figs. 1 and 2, and Additional file 2). The sequenced genomes of *P. patens* and *S. moellendorfii* contained only genes encoding for the ancestral BZR1-BAM and the progenitor of BAMs from clades I-III (Figs. 1, 2 and 6, and Additional file 2). Interestingly, orthologs of the other clades were found in the transcriptome of other bryophytes (Figs. 1 and 2, and Additional file 2). These findings indicate that at least some BAM clades were already present in the ancestor of all land plants, rather than emerging later as has been proposed previously [6]. Their absence in *P. patens* and *S. moellendorfii* may be the result of species-specific deletions, although it could also be caused by incomplete genome information, or assembly and annotation problems. On the other hand, green algae lacked clear orthologs of most clades identified in seed plants (Fig. 2 and Additional file 2). Taken together, our results suggest that the divergence of β-amylases clearly preceded the emergence of seed plants, but occurred after the colonization of terrestrial habitats (Fig. 8). The evolution of BZR-BAMs is complicated and previous studies have reached conflicting results [2, 5]. Attempts to elucidate their emergence are hampered by the scarcity of sequenced genomes from basal plants. Transcriptome data is an alternative, which we have used to fill the gaps, however the fragmentary nature of such sequences makes it difficult to establish whether a given BAM sequence contains a BZR1-like domain or not. Nonetheless we have found several sequences containing both a BZR-domain and a β-amylase domain in the transcriptomes of acrogymnosperms and ferns (Additional file 7). This places the fusion of these two domains before the emergence of the seed plants, rather than during the evolution of angiosperms as has been assumed previously [6]. Sequences of β-amylases similar to the BZR-BAMs are also present in bryophytes and lycophytes (Figs. 1 and 2), but we did not find any sequence containing both domains. It is unclear whether this reflects a genuine absence of such sequences as proposed by [5] or a limitation of the data used. We have tentatively placed the emergence of the BZR-BAM fusion proteins before the radiation of euphyllophytes, while the corresponding BAM domain was already present in bryophytes. Further work will be required to understand the function of these BAMs, and to determine if they contain a BZR-domain or not.

Sequences of full length BZR1-BAMs are present in ferns and gymnosperms (Fig. 2 and Additional file 2). However, and the functional brassinosteroid receptor BRI1 is only found in flowering plants [67]. It is interesting that the duplication of the BZR1-BAMs in flowering plants coincides with the emergence of these functional BRI1-receptors. However, in contrast to BZR1-BAMs, BRI1-like genes and other genes involved in brassinosteroid signaling are found in vascular plants other than angiosperms [68]. The emergence of BZR1-BAMs in ferns is consistent with a gradual emergence of the other components of the brassinosteroid signaling pathway during the evolution of vascular plants. The integration of metabolic signaling into brassinosteroid signaling and/or related signaling networks, as proposed for BZR1-BAMs [26], could have been advantageous even before the emergence of functional BRI1-receptors. Alternatively, the BZR-BAMs might have originated independently of the brassinosteroids and were only later recruited to the pathway. Further work will be required to determine the function of BZR1-BAMs in basal plants and their relation to brassinosteroids.

The presence of BZR-less sequences among BZR-BAMs is a puzzling feature of this clade. As these sequences are interspersed between BZR-BAMs throughout the evolution (Fig. 1) of vascular plants it appears that they either formed multiple times independently through the loss of the BZR domain, or conversely that the BZR-domain was acquired several times independently. A potential strategy to resolve this question would be to investigate the properties of these BZR-less BAMs. If they share the features of *At*BAM2 such as the formation of multimers and the dependence on potassium as a cofactor, this would support the hypothesis that they are related, and the BZR-domain was acquired multiple times. If on the other hand these features are unique to *At*BAM2, it is more likely that each BZR-less protein arose independently through the secondary loss of the BZR-domain.

The sequences of clades I, II and III from the seed plants form three strongly supported clades. Interestingly, in genomes and transcriptomes of all bryophytes, lycophytes and ferns, at least one BAM gene was found that clustered with these clades (Fig. 1 and Additional file 2). This could indicate that the three clades only diverged after the radiation of vascular plants, or that two orthologs have been lost in lycophytes and monilophytes. However, it is not possible to draw a final scenario due to the low signal in this part of the tree which hinders the resolution of the precise relationships between these three isoforms. In Arabidopsis, *At*BAM1 (Clade I) and *At*BAM3 (Clade III) have distinct functions. *At*BAM1 degrades starch in guard cells and in leaves during osmotic stress [20–23], while *At*BAM3 is responsible for night-time starch degradation in mesophyll cells [2]. Interestingly, while stomata are present in basal land plants, unequivocal active control of stomatal movements is only found in seed plants [69]. Stomata in ferns seem to close much more slowly, if at all [70–73]. It is possible that the recruitment of a β-amylase for stomatal carbon metabolism imposed conflicting selection pressures on the ancestral BAM, which could be resolved by a duplication event followed by isoform sub functionalization. It would be interesting to investigate the function of the ancestral BAM with regard to mesophyll and guard cell starch metabolism in ferns.

BAM clade IV, containing *At*BAM4 orthologs, is the least conserved amongst the eight identified clades. BAM4 orthologs were absent in over half of the analyzed species (Fig. 5 and Additional file 2). Given that BAM4 in Arabidopsis play an essential role for night-time starch degradation [2], our findings are surprising. We speculate that alternative pathways of starch degradation may exist among different flowering plants, which may be regulated by as-yet an unknown mechanism. *At*BAM4 can efficiently bind to starch. It was suggested that *At*BAM4 may work as a scaffold protein to facilitate the binding to starch of other glucan degrading enzymes [25]. If correct, enzymes normally interacting with BAM4 might have adapted to interact with starch directly in species where BAM4 was lost. Alternatively, it is possible that plants lacking BAM4 rely on other proteins to mediate the proposed interactions between starch and degrading enzymes. A potential candidate is the newly discovered BAM10, since it was also predicted to be catalytically inactive and to be localized to the plastid (Fig. 3, Table 2 and Additional file 4: Table S11). Circumstantially, the fact that while losses of either BAM4 or BAM10 were common amongst seed plants, but only three species (*Oryza brachyantha*, *Phyllostachys heterocycla* and *Picea abies*) lacked both proteins supports the hypothesis that both proteins have similar function. In tomato, BAM10 is widely expressed in starch synthesizing tissues (Additional file 5). BAM10 emerged before the radiation of seed plants, but it was lost in several species, including the model plant *A. thaliana*. The example of BAM4 and BAM10 highlights that insights gained from model plants, such as Arabidopsis, cannot always be translated to other species, and emphasizes the importance of molecular evolutionary studies to unravel the functional complexity of multigene families, such as the plant β-amylases.

In addition to the loss of BAM4 and the emergence of BAM10, our work uncovered the extensive amount of duplications that characterized the β-amylase gene family during the evolution of land plants. Over 60% of all analyzed species showed a duplication of at least one BAM gene (Additional file 3 and Additional file 2). Several duplications were even conserved across whole families, clearly indicating gene sub- or neo-functionalizations. In some cases, the duplication involved a shift in the localization of the proteins: both Brassicales and Poales carried two copy of BAMs from Clade V (BAM9), and in both families one isoform was predicted to be localized to the plastid, while the other was predicted to be cytosolic (Additional file 4: Table S10). The conservation of duplicated copies of the same BAM isoform in many plant lineages may reflect the potential evolutionary advantage of having plasticity and flexibility in the starch degrading pathways. The detailed picture provided here opens new possibilities for investigating the importance of starch degradation in an evolutionary context.

## Conclusions

We identified 961 β-amylase sequences from 136 different algae and land plant species and reconstructed their evolutionary history. Our comprehensive phylogenetic analyses reveal that extensive duplications of many β-amylase genes during the evolution and diversification of land plants led to an increase in the overall number of BAM genes and promoted substantial sub- or neo-functionalization amongst the different members of the family. This study provides essential insights for future molecular evolution and functional studies of this important class of glucan hydrolases and regulatory proteins.

## Additional files

Additional file 1: **Table S1.** List of the species queried for the phylogenetic analysis and the corresponding sequences sources. (PDF 122 kb)

Additional file 2: Taxonomy of the species used in this study and list of the accession numbers of the BAM sequences used for the phylogenetic analysis. (XLSX 115 kb)

Additional file 3: Copy number variations of *BAM* genes in the analyzed land plant species. (PDF 267 kb)

Additional file 4: **Table S2-S11.** Prediction of BAM isoforms subcellular localization. (PDF 470 kb)

Additional file 5: *SolycBAM10* gene is expressed in most plant tissues. (PDF 193 kb)

Additional file 6: Protein alignment of BAM isoforms from Arabidopsis and BAM10 from *Theobroma cacao*. (PDF 1188 kb)

Additional file 7: Protein alignment of BZR1-domain containing β-amylases from representative non-flowering plants. (PDF 159 kb)

## Abbreviations

BAM: β-Amylase
BES1: BRI1-EMS-SUPPRESSOR 1
BRI1: BRASSINOSTEROID INSENSITIVE 1
BZR1: BRASSINAZOLE RESISTANT 1
BZR1: Brassinazole Resistant 1
Glu: Glutamic acid
*sex*: Starch excess
Thr: Threonine
