# Supplementary material for: The evolution of functional complexity within the β-amylase gene family in land plants
Source: BMC Evol Biol. 2019 Feb 28;19:66. doi: 10.1186/s12862-019-1395-2 (PMC6394054; doi:10.1186/s12862-019-1395-2)
Supplement: Supplementary file 1 — Table S1. List of the species queried for the phylogenetic analysis and the corresponding sequences sources. (PDF 122 kb) [file 12862_2019_1395_MOESM1_ESM.pdf]

Additional file 1: Table S1. List of the species queried for the phylogenetic analysis and the corresponding sequences sources.

Botanical name	Common name	Source	Website
Abies lasiocarpa	Rocky Mountain fir	Transcriptome	oneKP
Agathis robusta	Queensland kauri pine	Transcriptome	oneKP
Amborella trichopoda		Genome	NCBI blast
Angiopteris evecta	Giant fern	Transcriptome	oneKP
Aquilegia coerulea	Colorado blue Columbine	Genome	Phytozome
Arabidopsis lyrata		Genome	NCBI blast
Arabidopsis thaliana	Thale cress	Genome	NCBI blast
Arachis duranensis		Genome	http://www.peanutbase.org/
Arachis ipaensis		Genome	http://www.peanutbase.org/
Atrichum angustatum		Transcriptome	oneKP
Beta vulgaris	Sugar beet	Genome	NCBI blast
Brachypodium distachyon	Purple false brome	Genome	NCBI blast
Brassica napus	Rapeseed	Genome	NCBI blast
Brassica rapa	Field mustard	Genome	NCBI blast
Camelina sativa	False flax	Genome	NCBI blast
Camellia sinensis	Tea plant	Genome	NCBI blast
Capsella rubella	Pink shepherd’s purse	Genome	NCBI blast
Capsicium annuum	Chilli	Genome	http://peppersequence.genomics.cn/page/species/index.jsp
Carica papaya	Papaya	Genome	Phytozome
Cathaya argyrophylla		Transcriptome	oneKP
Cedrus libani	Lebanon cedar	Transcriptome	oneKP
Cicer arietinum	Chickpea	Genome	NCBI blast
Citrullus lanatus	Water melon	Genome	http://www.icugi.org
Citrus clementia	Clementine	Genome	NCBI blast
Citrus sinensis	Sweet orange	Genome	NCBI blast
Coffea canephora	Coffee	Genome	NCBI blast
Coleochaete irregularis		Transcriptome	oneKP
Coleochaete scutata		Transcriptome	oneKP
Cucumis melo	Muskmelon	Genome	NCBI blast
Cucumis sativus	Cucumber	Genome	NCBI blast
Culcita macrocarpa		Transcriptome	oneKP
Cupressus dupreziana	Moroccan cypress	Transcriptome	oneKP
Cycas micholitzii		Transcriptome	oneKP
Cynara cardunculus	Artichoke	Genome	NCBI blast
Daucus carota	Carrot	Genome	NCBI blast
Dion edule	Chestnut Dion	Transcriptome	oneKP
Elaeis guineesis	African oil palm	Genome	NCBI blast
Encephalartos barteri		Transcriptome	oneKP
Ephedra sinica	Ma Huang	Transcriptome	oneKP
Equisetum diffusum	Himalayan horsetail	Transcriptome	oneKP
Equisetum hyemale	Rough horsetail	Transcriptome	oneKP
Erythranthe guttatus	Seep Monkeyflower	Genome	NCBI blast

Eucalyptus grandis	Rose gum	Genome	NCBI blast
Eutrema salsuginea	Saltwater cress	Genome	NCBI blast
Fragaria Vesca	Strawberry	Genome	NCBI blast
Ginkgo biloba	Maidenhair tree	Transcriptome	oneKP
Glycine max	Soybean	Genome	NCBI blast
Gnetum montanum		Transcriptome	oneKP
Gossypium raimondii	Cotton	Genome	NCBI blast
Griselinia racemosa		Transcriptome	oneKP
Hordeum vulgare	Barley	Genome	NCBI blast
Huperzia lucidula	Shining firmoss	Transcriptome	oneKP
Huperzia myrsinites		Transcriptome	oneKP
Ilex vomitoria	Yaupon holly	Transcriptome	oneKP
Jatropha curcas	Barbados nut	Genome	NCBI blast
Juniperus scopulorum	Rocky Mountain juniper	Transcriptome	oneKP
Keteleeria evelyniana		Transcriptome	oneKP
Leontopodium alpinum	Edelweiss	Transcriptome	oneKP
Linum usitatissimum	Common flax	Genome	Phytozome
Malus domestica	Apple	Genome	NCBI blast
Manihot esculenta	Cassava	Genome	Phytozome
Marchantia paleacea		Transcriptome	oneKP
Marchantia polymorpha	Umbrella liverwort	Transcriptome	oneKP
Medicago truncatula	Barrelclover	Genome	NCBI blast
Morus notabilis	Mulberry	Genome	NCBI blast
Musa acuminata	Banana	Genome	NCBI blast
Nageia nagi	Asian bayberry	Transcriptome	oneKP
Nelumbo nucifera	Sacred lotus	Genome	NCBI blast
Nicotiana benthamiana	Tobacco	Genome	http://sydney.edu.au/science/molecular_bioscience/sites/benthamiana/
Nothoceros aenigmaticus		Transcriptome	oneKP
Nothoceros vincentianus		Transcriptome	oneKP
Ophioglossum vulgatum	Southern adders-tongue	Transcriptome	oneKP
Oryza brachyantha		Genome	NCBI blast
Oryza sativa	Rice	Genome	NCBI blast
Panicum virgatum	Switchgrass	Genome	Phytozome
Phaseolus vulgaris	Common bean	Genome	NCBI blast
Phoenix dactylifera	Date palm	Genome	NCBI blast
Phyllostachys heterocycla	Moso bamboo	Genome	http://202.127.18.221/bamboo/down.php
Physcomitrella patens	Spreading earthmoss	Genome	NCBI blast
Picea abies	Norway spruce	Genome	http://congenie.org/start
Picea engelmandii	White spruce	Transcriptome	oneKP
Pinus radiata	Monterey pine	Transcriptome	oneKP
Pinus taeda	Loblolly pine	Genome	http://dendrome.ucdavis.edu/resources/blast/
Podocarpus coriaceus	Yucca plum pine	Transcriptome	oneKP
Podocarpus rubens		Transcriptome	oneKP
Populus trichocarpa	California poplar	Genome	NCBI blast
Prunus mume	Mei	Genome	NCBI blast

Prunus persica	Peach	Genome	NCBI blast
Pseudolarix amabilis	Golden larch	Transcriptome	oneKP
Pseudotsuga wilsoniana	Chinese Douglas fir	Transcriptome	oneKP
Psilotum nudum	Skeleton fork fern	Transcriptome	oneKP
Ricinus Communis	Castorbean	Genome	NCBI blast
Salix purpurea	Purple willow	Genome	Phytozome
Sciadoptys verticillata	Japanese umbrella-pine	Transcriptome	oneKP
Selaginella acanthonota		Transcriptome	oneKP
Selaginella kraussiana	African clubmoss	Transcriptome	oneKP
Selaginella moellendorffii		Genome	NCBI blast
Sequoiadendron giganteum	Giant sequoia	Transcriptome	oneKP
Sesamum indicum	Sesame	Genome	NCBI blast
Setaria italica	Foxtail millet	Genome	NCBI blast
Solanum lycopersicum	Tomato	Genome	NCBI blast
Solanum tuberosum	Potato	Genome	NCBI blast
Sorghum bicolor	Sorghum	Genome	NCBI blast
Spirodela polyrhiza	Common duckweed	Genome	pgir.rutgers.edu/blast/blast.cgi
Spirogyra sp	Water silk	Transcriptome	oneKP
Stangeria eriopus		Transcriptome	oneKP
Taiwania cryptomerioides		Transcriptome	oneKP
Takakia lepidozioides		Transcriptome	oneKP
Tarenaya hassleriana	Spider flower	Genome	NCBI blast
Taxus baccata	European yew	Transcriptome	oneKP
Theobroma cacao	Cacao	Genome	NCBI blast
Torreya nucifera	Japanese nutmeg-yew	Transcriptome	oneKP
Treubia lacunosa		Transcriptome	oneKP
Tsuga heterophylla	Western Hemlock	Transcriptome	oneKP
Vitis vinifera	Grape	Genome	NCBI blast
Welwitschia mirabilis		Transcriptome	oneKP
Wollemia nobilis	Wollemi pine	Transcriptome	oneKP
Zea mays	Maize	Genome	NCBI blast

(https://phytozome.jgi.doe.gov/pz/portal.html) Phytozome
(https://www.bioinfodata.org/Blast4OneKP/) oneKP
(https://blast.ncbi.nlm.nih.gov/Blast.cgi), NCBI blast
Additional databases are listed within the table.
