# Supplementary material for: The evolution of functional complexity within the β-amylase gene family in land plants
Source: BMC Evol Biol. 2019 Feb 28;19:66. doi: 10.1186/s12862-019-1395-2 (PMC6394054; doi:10.1186/s12862-019-1395-2)
Supplement: Supplementary file 3 — Copy number variations of BAM genes in the analyzed land plant species. (PDF 267 kb) [file 12862_2019_1395_MOESM3_ESM.pdf]

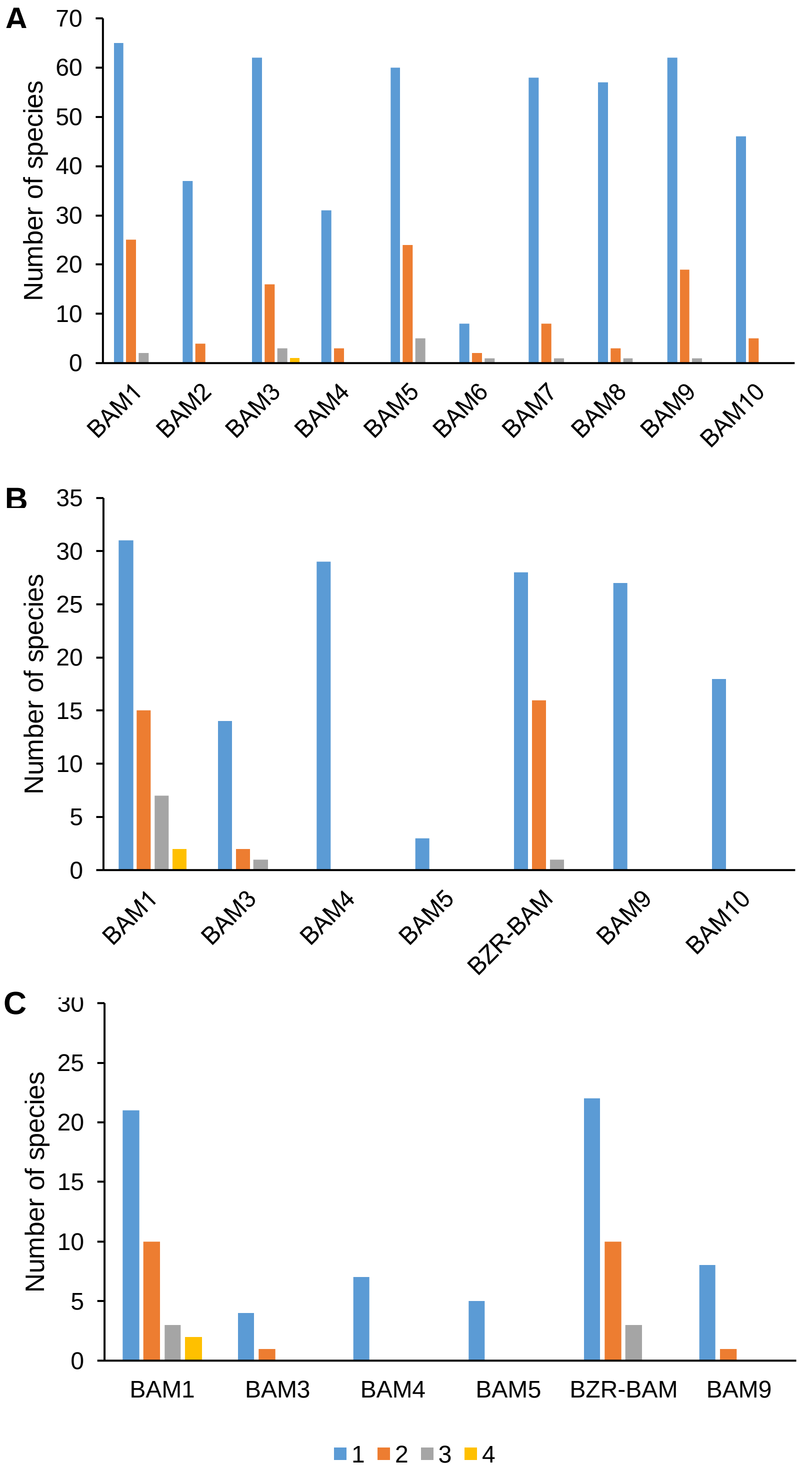

Additional file 3. Copy number variations of *BAM* genes in the analyzed land plant species. The graphs indicate the number of species containing at least "n" (n = 1 to 4) copies of β-amylase isoforms in angiosperms (A), gymnosperms (B), and basal land plant lineages (C). Note that the number of BAM isoforms increased across the evolution of land plants. The identity of the BAM sequences was determined using the phylogenetic relationship depicted in Figure 1.
