# Supplementary material for: The evolution of functional complexity within the β-amylase gene family in land plants
Source: BMC Evol Biol. 2019 Feb 28;19:66. doi: 10.1186/s12862-019-1395-2 (PMC6394054; doi:10.1186/s12862-019-1395-2)
Supplement: Supplementary file 4 — Table S2-S11. Prediction of BAM isoforms subcellular localization. (PDF 470 kb) [file 12862_2019_1395_MOESM4_ESM.pdf]

Table S2. Predicted BAM1 orthologs subcellular localization.

Name	Length	Score	cTP	CS-score	cTP-length
BAM1.1_*A.robusta*_MIXZ2012495	617	0.53	Y	-1.61	40
BAM1.1_*B.distachyon*_XP_003558837.1	573	0.583	Y	4.748	39
BAM1.1_*B.napus*_XP_013747707.1	570	0.57	Y	5.589	74
BAM1.1_*B.rapa*_XP_009102529.1	570	0.57	Y	5.589	74
BAM1.1_*C.arietinum*_XP_004515248.1	573	0.54	Y	12.047	31
BAM1.1_*C.melo*_XP_008438436.1	577	0.543	Y	2.009	49
BAM1.1_*C.sativus*_XP_004134029.1	577	0.527	Y	2.465	34
BAM1.1_*Camelina_sativa*_XP_010488521.1	575	0.548	Y	4.618	67
BAM1.1_*F.vesca*_XP_004296549.1	578	0.547	Y	9.41	34
BAM1.1_*G.max*_XP_003534086.1	569	0.521	Y	4.776	69
BAM1.1_*G.raimondii*_XP_012454771.1	587	0.509	Y	3.219	29
BAM1.1_*H.vulgare*_BAJ96121.1	551	0.54	Y	6.994	30
BAM1.1_*M.domestica*_XP_008391283.1	571	0.549	Y	6.728	80
BAM1.1_*M.esculenta*_cassava4.1_004325m	582	0.531	Y	13.835	34
BAM1.1_*N.benthamiana*_Nbv5tr6236522	576	0.516	Y	5.069	80
BAM1.1_*N.nucifera*_XP_010263970.1	568	0.536	Y	4.346	71
BAM1.1_*O.sativa*_NP_001048926.1	557	0.542	Y	1.751	38
BAM1.1_*P.coriaceus*_SCEB2055235	620	0.519	Y	5.627	13
BAM1.1_*P.dactylifera*_XP_008775132.1	572	0.451	-	2.793	69
BAM1.1_*P.engelmandii*_AWQB200193	623	0.478	-	2.195	76
BAM1.1_*P.rubens*_BAM1.1_XLGK2007963	621	0.528	Y	5.627	13
BAM1.1_*P.taeda*_PITA_000025218RA	623	0.451	-	1.035	13
BAM1.1_*P.trichocarpa*_XP_002311706.1	562	0.506	Y	5.628	62
BAM1.1_*P.vulgaris*_XP_007152599.1	568	0.54	Y	5.977	68
BAM1.1_*S.bicolor*_XP_002468533.1	564	0.565	Y	3.825	55
BAM1.1_*S.indicum*_XP_011091372.1	580	0.547	Y	2.879	70
BAM1.1_*S.italica*_XP_004985750.1	563	0.563	Y	5.856	79
BAM1.1_*S.lycopersicum*_NP_001234556.2	580	0.538	Y	5.069	84
BAM1.1_*S.purpurea*_SapurV1A.0021s0270	582	0.519	Y	12.613	30
BAM1.1_*S.tuberosum*_XP_006340896.1	579	0.541	Y	3.915	85
BAM1.1_*T.heterophylla*_GAMH2004527	611	0.447	-	0.85	64
BAM1.1_*V.vinifera*_XP_002285569.1	573	0.491	-	11.114	34
BAM1.1_*Z.mays*_NP_001147532.1	544	0.58	Y	10.628	63
BAM1.2_*B.distachyon*_XP_003571854.1	534	0.588	Y	13.063	59
BAM1.2_*B.napus*_XP_013750064.1	564	0.576	Y	5.589	68
BAM1.2_*B.rapa*_XP_009135884.1	564	0.543	Y	5.589	70
BAM1.2_*C.melo*_XP_008460711.1	545	0.549	Y	7.246	72
BAM1.2_*C.sativus*_XP_004147196.1	545	0.563	Y	7.246	72
BAM1.2_*G.max*_XP_003548316.1	575	0.548	Y	4.624	75

BAM1.2_*M.domestica*_XP_008342553.1	571	0.551	Y	5.208	80
BAM1.2_*M.esculenta*_cassava4.1_004345m	581	0.522	Y	6.559	75
BAM1.2_*N.benthamiana*_Nbv5tr6214720	565	0.536	Y	4.153	48
BAM1.2_*O.sativa*_NP_001064798.1	535	0.581	Y	9.594	62
BAM1.2_*P.heterocycla*_PH01000560G0630	449	0.483	-	5.868	31
BAM1.2_*P.taeda*_PITA_000025216RA	620	0.463	-	2.333	77
BAM1.2_*P.trichocarpa*_XP_002314522.2	586	0.465	-	4.905	45
BAM1.2_*S.bicolor*_XP_002467119.1	547	0.584	Y	7.393	64
BAM1.2_*S.indicum*_XP_011073736.1	583	0.558	Y	3.163	82
BAM1.2_*S.purpurea*_SapurV1A.0096s0290	584	0.461	-	5.628	82
BAM1.2_*Z.mays*_NP_001148159.1	573	0.556	Y	10.374	26
BAM1.3_*C.sativa*_XP_010513371.1	576	0.554	Y	4.624	75
BAM1.3_*G.raimondii*_XP_012484484.1	589	0.534	Y	2.763	66
BAM1.3_*N.nagi*_UUJS2117213	620	0.507	Y	5.627	13
BAM1.3_*N.nucifera*_XP_010264799.	594	0.5	-	13.774	33
BAM1.3_*P.heterocycla*_PH01000010G113	484	0.48	-	2.921	44
BAM1.3_*P.taeda*_PITA_000025217RA	623	0.446	-	7.149	69
BAM1.4_*P.taeda*_PITA_000025219RA	580	0.528	Y	7.031	46
BAM1_*A.corulea*_Aquca_025_00081	595	0.535	Y	5.186	36
BAM1_*A.ipaensis*_Araip.183TE	519	0.555	Y	2.992	68
BAM1_*A.lasiocarpa*_VSRH2002636	622	0.456	-	2.195	76
BAM1_*A.lyrata*_XP_002885629.1	572	0.53	Y	5.661	40
BAM1_*A.thaliana*_NP_189034.1	575	0.524	Y	5.661	41
BAM1_*A.trichopoda*_XP_006851336.1	587	0.541	Y	8.354	36
BAM1_*B.vulgaris*_XP_010666969.1	579	0.528	Y	6.763	101
BAM1_*C.annum*_Capana03g004414	577	0.516	Y	4.86	41
BAM1_*C.argyrophylla*_NPRL2008412	622	0.451	-	6.203	101
BAM1_*C.canephora*_CDP20299.1	582	0.525	Y	10.925	73
BAM1_*C.cardunculus*_KVH91041.1	572	0.54	Y	7.816	65
BAM1_*C.clementia*_XP_006420416.1	580	0.494	-	7.65	90
BAM1_*C.lantus*_Cla007635	554	0.571	Y	11.184	73
BAM1_*C.papaya*_evm.TU.supercontig_59.40	574	0.548	Y	6.253	33
BAM1_*C.rubella*_XP_006296695.1	573	0.535	Y	5	71
BAM1_*Camellia.sinensis*_AKQ62956.1	581	0.538	Y	5.895	47
BAM1_*Citrus.sinensis*_XP_006493994.1	580	0.498	-	7.65	90
BAM1_*D.carota*_KZM87169.1	569	0.504	Y	6.861	45
BAM1_*E.grandis*_XP_010023784.1	584	0.492	-	13.835	34
BAM1_*E.guineesis*_XP_010918964.1	571	0.454	-	10.873	29
BAM1_*E.guttatus*_XP_012829096.1	574	0.563	Y	6.906	98
BAM1_*E.salsuginea*_XP_006418770.1	582	0.539	Y	7.111	79
BAM1_*G.racemosa*_QIKZ2005472	454	0.49	-	7.816	70
BAM1_*I.vomitoria*_ASMV2109428	577	0.505	Y	5.069	79
BAM1_*J.curcas*_XP_012077650.1	583	0.489	-	6.203	75

BAM1_*L.alpinum*_DOVJ2001331	572	0.511	Y	7.816	70
BAM1_*M.acuminata*_XP_009400488.1	590	0.522	Y	4.175	79
BAM1_*M.notabilis*_XP_010111574.1	604	0.553	Y	0.627	84
BAM1_*M.trunculata*_XP_013452815.1	572	0.51	Y	4.772	44
BAM1_*O.brachyantha*_XP_006662424.1	453	0.451	-	1.567	60
BAM1_*P.abies*_MA_124514g0010	624	0.479	-	4.012	55
BAM1_*P.mume*_XP_008224054.1	569	0.555	Y	4.173	77
BAM1_*R.communis*_XP_002518196.1	574	0.494	-	7.963	78
BAM1_*S.polyrhiza*_Spipo16G0046000	575	0.453	-	3.779	81
BAM1_*T.hassleriana*_XP_010550849.1	591	0.513	Y	13.302	83

Table S3. Predicted BAM2 orthologs subcellular localization.

Name	Length	Score	cTP	CS-score	cTP-length
BAM2.1_*B.napus*_XP_013660393.1	533	0.532	Y	6.354	23
BAM2.1_*C.sativa*_XP_010427089.1	554	0.559	Y	1.929	51
BAM2.1_*P.dactylifera*_XP_008787503.1	534	0.566	Y	2.459	57
BAM2_*V.vinifera*_XP_002274612.2	554	0.583	Y	3.939	61
BAM2.2_*B.napus*_XP_013731270.1	539	0.526	Y	6.215	48
BAM2.2_*C.sativa*_XP_010456279.1	555	0.564	Y	1.929	50
BAM2.2_*P.dactylifera*_XP_008804014.1	548	0.552	Y	3.861	62
BAM2_*A.lyrata*_XP_002875024.1	542	0.563	Y	5.840	56
BAM2_*A.thaliana*_NP_191958.3	542	0.557	Y	5.840	55
BAM2_*B.distachyon*_XP_010232924.1	530	0.432	-	2.404	89
BAM2_*B.rapa*_XP_009111477.1	541	0.53	Y	3.035	48
BAM2_*B.vulgaris*_XP_010670423.1	574	0.578	Y	4.919	55
BAM2_*C.annum*_Capana08g000914	433	0.449	-	5.558	16
BAM2_*C.clementia*_XP_006445046.1	562	0.555	Y	-0.819	66
BAM2_*C.papaya*_evm.TU.supercontig_18.253	504	0.574	Y	1.152	45
BAM2_*C.rubella*_XP_006287426.1	549	0.553	Y	5.533	49
BAM2_*C.sinensis*_AHC32020.1	556	0.575	Y	2.422	60
BAM2_*Citrus.sinensis*_XP_006491095.1	562	0.555	Y	-0.819	66
BAM2_*E.grandis*_XP_010055131.1	546	0.561	Y	9.724	67
BAM2_*E.guineesis*_XP_010934793.1	553	0.58	Y	1.456	46
BAM2_*E.guttatus*_XP_012843727.1	558	0.538	Y	4.775	55
BAM2_*E.salsuginea*_XP_006396247.1	546	0.552	Y	1.929	52
BAM2_*F.vesca*_XP_004306786.1	544	0.538	Y	1.217	10
BAM2_*G.raimondii*_XP_012489942.1	536	0.54	Y	6.041	55
BAM2_*J.curcas*_XP_012083395.1	537	0.548	Y	-0.221	46
BAM2_*M.acuminata*_XP_009392820.1	541	0.523	Y	2.448	21
BAM2_*M.domestica*_XP_008338858.1	548	0.562	Y	4.418	74
BAM2_*M.esculenta*_cassava4.1_022883m	540	0.576	Y	6.740	58
BAM2_*M.notabilis*_XP_010105936.1	554	0.586	Y	5.457	36
BAM2_*O.sativa*_NP_001063976.1	533	0.573	Y	0.678	40
BAM2_*P.mume*_XP_008232901.1	538	0.582	Y	8.237	71
BAM2_*P.trichocarpa*_XP_002320794.2	539	0.569	Y	3.519	37
BAM2_*R.communis*_XP_0	609	0.576	Y	7.687	45
BAM2_*S.indicum*_XP_01	527	0.542	Y	8.744	52
BAM2_*S.italica*_XP_012699441.1	566	0.548	Y	2.474	17
BAM2_*T.cacao*_XP_007051810.1	554	0.568	Y	2.583	48
BAM2_*T.hassleriana*_XP_010540283.1	550	0.512	Y	3.043	20

Table S4. Predicted BAM3 orthologs subcellular localization.

Name	Length	Score	cTP	CS-score	cTP-length
BAM3.1_*B.napus*_XP_013721634.1	549	0.515	Y	1.424	48
BAM3.1_*B.rapa*_XP_009136825.1	548	0.51	Y	1.424	48
BAM3.1_*C.arietinum*_XP_004511752.1	545	0.447	-	1.775	63
BAM3.1_*C.sativa*_XP_010440058.1	548	0.519	Y	1.424	49
BAM3.1_*E.guttatus*_XP_012829112.1	553	0.512	Y	3.481	42
BAM3.1_*G.max*_XP_006573703.1	548	0.5	Y	3.437	43
BAM3.1_*G.raimondii*_XP_012488822.1	539	0.492	-	1.138	54
BAM3.1_*M.acuminata*_XP_009397011.1	547	0.526	Y	9.827	44
BAM3.1_*M.trunculata*_XP_003611408.1	543	0.444	-	-0.289	32
BAM3.1_*P.abies*_MA_129283g0010	552	0.525	Y	-1.768	49
BAM3.1_*P.taeda*_PITA_000032112RA	557	0.525	Y	4.899	2
BAM3.1_*P.trichocarpa*_XP_006385389.1	547	0.495	-	5.345	40
BAM3.1_*S.lycopersicum*_XP_004244551.1	542	0.45	-	-1.196	13
BAM3.1_*S.purpurea*_SapurV1A.0380s0080	547	0.513	Y	7.486	40
BAM3.1_*S.tuberosum*_XP_006362484.1	541	0.466	-	5.338	39
BAM3.1_*Z.mays*_XP_008658990.1	553	0.519	Y	9.935	48
BAM3.2_*B.napus*_XP_013737190.1	548	0.51	Y	1.424	48
BAM3.2_*B.rapa*_XP_009144721.1	549	0.515	Y	1.424	48
BAM3.2_*C.arietinum*_XP_004508980.1	545	0.468	-	5.353	58
BAM3.2_*C.sativa*_XP_010449668.1	548	0.524	Y	1.424	49
BAM3.2_*D.carota*_KZN07474.1	545	0.53	Y	5.120	31
BAM3.2_*E.guttatus*_XP_012852342.1	553	0.517	Y	3.481	42
BAM3.2_*G.max*_NP_001236350.1	540	0.529	Y	3.295	45
BAM3.2_*G.raimondii*_XP_012439399.1	508	0.513	Y	4.235	42
BAM3.2_*M.acuminata*_XP_009413253.1	549	0.485	-	-1.056	45
BAM3.2_*M.trunculata*_XP_003611409.1	543	0.473	-	2.905	63
BAM3.3_*M.trunculata*_XP_013457558.1	541	0.47	-	3.687	54
BAM3.2_*P.abies*_MA_3193g0010	552	0.507	Y	-0.356	49
BAM3.2_*P.taeda*_PITA_000045784RA	557	0.511	Y	2.237	53
BAM3.2_*P.trichocarpa*_XP_006385589.1	548	0.512	Y	5.345	40
BAM3.2_*S.lycopersicum*_XP_004245844.1	546	0.497	-	5.289	41
BAM3.2_*S.purpurea*_SapurV1A.0241s0110	552	0.481	-	2.917	32
BAM3.2_*S.tuberosum*_NP_001275172.1	545	0.477	-	5.289	40
BAM3.2_*Z.mays*_XP_008658465.1	553	0.519	Y	9.935	48
BAM3.3_*C.sativa*_XP_010434720.1	548	0.519	Y	1.424	49
BAM3.3_*G.max*_XP_003539125.1	554	0.49	-	3.437	43
BAM3.3_*P.taeda*_PITA_000047084RA	593	0.497	-	0.683	23
BAM3.4_*G.max*_XP_003524296.1	547	0.503	Y	3.920	40
BAM3_*A.corulea*_Aquca_053_00137	548	0.538	Y	1.306	40

BAM3_*A.ipaensis*_Araip.67F6M	564	0.487	-	2.325	64
BAM3_*A.lyrata*_XP_002868085.1	548	0.521	Y	1.424	49
BAM3_*A.thaliana*_NP_567523.1	548	0.518	Y	1.424	49
BAM3_*A.trichopoda*_XP_011621487.1	552	0.478	-	2.384	43
BAM3_*B.distachyon*_XP_003574353.1	548	0.505	Y	7.257	45
BAM3_*B.vulgaris*_XP_010695452.1	546	0.452	-	6.100	16
BAM3_*C.annum*_Capana01g000793	545	0.509	Y	5.289	40
BAM3_*C.canephora*_CDP13430.1	547	0.531	Y	1.862	42
BAM3_*C.cardunculus*_KVH95266.1	556	0.487	-	5.009	56
BAM3_*C.clementia*_XP_006440139.1	551	0.522	Y	5.478	66
BAM3_*C.lantus*_Cla008766	537	0.449	-	2.048	46
BAM3_*C.melo*_XP_008448759.1	537	0.439	-	2.048	46
BAM3_*C.rubella*_XP_006285145.1	548	0.527	Y	1.424	49
BAM3_*C.sativus*_XP_004147264.1	538	0.442	-	2.048	46
BAM3_*Camellia.sinensis*_AHJ09602.1	548	0.53	Y	2.756	41
BAM3_*Citrus.sinensis*_XP_006477060.1	551	0.522	Y	5.478	66
BAM3_*E.grandis*_XP_010055392.1	543	0.494	-	2.550	42
BAM3_*E.guineesis*_XP_010919815.1	546	0.464	-	0.367	23
BAM3_*E.salsuginea*_XP_006414272.1	548	0.526	Y	1.424	48
BAM3_*F.vesca*_XP_004300297.1	553	0.516	Y	5.120	34
BAM3_*G.racemosa*_QIKZ2022040	538	0.471	-	1.630	34
BAM3_*H.vulgare*_BAJ90222.1	549	0.496	-	7.257	47
BAM3_*I.vomitoria*_ASMV2109425	542	0.502	Y	1.516	40
BAM3_*J.curcas*_XP_012075356.1	547	0.488	-	0.606	32
BAM3_*L.alpinum*_DOVJ2007219	468	0.446	-	-5.321	2
BAM3_*M.domestica*_XP_008361217.1	547	0.477	-	4.715	40
BAM3_*M.esculenta*_cassava4.1_034006m	572	0.476	-	3.151	42
BAM3_*M.notabilis*_XP_010110537.1	544	0.472	-	5.707	45
BAM3_*N.benthamiana*_Nbv5tr6221381	548	0.459	-	2.349	42
BAM3_*N.nucifera*_XP_010274550.1	547	0.513	Y	2.670	43
BAM3_*O.brachyantha*_XP_006662612.1	305	0.506	Y	3.821	53
BAM3_*O.sativa*_NP_001065418.2	522	0.513	Y	6.307	45
BAM3_*P.dactylifera*_XP_008794866.1	547	0.507	Y	8.529	46
BAM3_*P.mume*_XP_008229498.1	547	0.461	-	4.715	40
BAM3_*P.persica*_XP_007209867.1	547	0.481	-	4.715	40
BAM3_*P.vulgaris*_XP_007155732.1	548	0.46	-	4.468	63
BAM3_*R.communis*_XP_002517513.1	547	0.497	-	3.878	40
BAM3_*S.bicolor*_XP_002464915.1	557	0.509	Y	8.636	53
BAM3_*S.indicum*_XP_011070282.1	549	0.519	Y	5.168	38
BAM3_*S.italica*_XP_004983616.1	557	0.527	Y	13.487	54
BAM3_*T.cacao*_XP_007039629.1	575	0.576	Y	2.962	59
BAM3_*T.hassleriana*_XP_010531694.1	549	0.5	-	3.595	44
BAM3_*V.vinifera*_XP_002282871.1	543	0.509	Y	3.869	41

Table S5. Predicted BAM4 orthologs subcellular localization.

Name	Length	Score	cTP	CS-score	cTP-length
BAM4.1_*B.napus*_XP_013685544.1	523	0.57	Y	3.653	78
BAM4.1_*B.rapa*_XP_009132357.1	532	0.532	Y	0.433	61
BAM4.1_*C.sativa*_XP_010483031.1	531	0.533	Y	2.467	86
BAM4.2_*B.napus*_XP_013676174.1	528	0.529	Y	3.653	83
BAM4.2_*B.rapa*_XP_009126986.1	528	0.516	Y	3.653	83
BAM4.2_*C.sativa*_XP_010450090.1	531	0.542	Y	2.467	86
BAM4_*A.corulea*_Aquca_005_00321	528	0.55	Y	3.932	17
BAM4_*A.lyrata*_XP_002864407.1	531	0.539	Y	2.467	86
BAM4_*A.thaliana*_NP_568829.2	531	0.541	Y	2.600	62
BAM4_*B.vulgaris*_XP_010675936.1	524	0.472	-	0.385	76
BAM4_*C.cardunculus*_KVH99190.1	574	0.482	-	4.998	57
BAM4_*C.lantus*_Cla004462	527	0.526	Y	1.593	57
BAM4_*C.melo*_XP_008449033.1	524	0.51	Y	1.656	62
BAM4_*C.papaya*_evm.model.supercontig_12.148	431	0.541	Y	-0.993	72
BAM4_*C.rubella*_XP_006280293.1	531	0.535	Y	2.600	62
BAM4_*C.sativus*_XP_004148285.1	520	0.56	Y	1.965	61
BAM4_*C.sinensis*_AKQ62957.1	518	0.461	-	5.086	72
BAM4_*E.salsuginea*_XP_006401422.1	531	0.526	Y	-2.824	33
BAM4_*F.vesca*_XP_004291809.1	516	0.551	Y	4.784	80
BAM4_*G.raimondii*_XP_012473201.1	518	0.534	Y	1.593	58
BAM4_*J.curcas*_XP_012071010.1	521	0.495	-	2.054	75
BAM4_*L.alpinum*_DOVJ2015003	495	0.533	Y	5.932	40
BAM4_*M.domestica*_XP_008366443.1	525	0.515	Y	3.843	12
BAM4_*M.esculenta*_cassava4.1_005532m	522	0.535	Y	-1.003	14
BAM4_*N.nucifera*_XP_010270251.1	447	0.465	-	2.873	73
BAM4_*P.mume*_XP_008227162.1	521	0.479	-	6.111	10
BAM4_*P.taeda*_PITA_000022542RA	574	0.531	Y	2.178	96
BAM4_*T.cacao*_XP_007020502.1	521	0.469	-	1.593	61
BAM4_*T.hassleriana*_XP_010536726.1	531	0.537	Y	-0.249	62
BAM4_*V.vinifera*_XP_002265698.1	522	0.452	-	1.472	85

Table S6. Predicted BAM5 orthologs subcellular localization.

Name	Length	Score	cTP	CS-score	cTP-length
BAM5.1_*B.napus*_XP_013655702.1	498	0.433	-	1.783	26
BAM5.1_*B.rapa*_XP_009107836.1	498	0.433	-	1.783	26
BAM5.1_*C.sativa*_XP_010435035.1	498	0.431	-	1.783	26
BAM5.1_*Z.mays*_NP_001105496.1	488	0.434	-	4.366	34
BAM5.2_*B.napus*_XP_013653901.1	520	0.427	-	-4.577	23
BAM5.2_*B.rapa*_XP_009106879.1	498	0.427	-	-4.577	23
BAM5.2_*C.sativa*_XP_010440324.1	498	0.433	-	1.783	26
BAM5.2_*Z.mays*_NP_001168436.1	595	0.572		4.627	49
BAM5.3_*C.sativa*_XP_010449959.1	498	0.433	-	1.783	26
BAM5.1_*B.vulgaris*_XP_010688831.1	571	0.515	Y	0.191	47
BAM5.1_*C.annum*_Capana07g001521	572	0.501	Y	3.879	64
BAM5.1_*C.arietinum*_XP_004487367.1	595	0.438	-	-0.926	5
BAM5.1_*E.guttatus*_XP_012852439.1	584	0.53	Y	3.078	70
BAM6.1_*G.max*_XP_003539882.1	496	0.438	-	1.195	25
BAM5.1_*G.raimondii*_XP_012434830.1	492	0.431	-	2.403	8
BAM5.1_*H.vulgare*_BAA04815	535	0.436	-	3.357	6
BAM5.1_*M.acuminata*_XP_009388866.1	503	0.441	-	5.838	27
BAM5.1_*M.trunculata*_XP_013455524.1	496	0.436	-	0.482	25
BAM5.1_*O.brachyantha*_XP_006657803.1	488	0.438	-	3.903	6
BAM5.1_*P.heterocycla*_PH01000656G0560	488	0.433	-	5.951	34
BAM5.1_*S.italica*_XP_004957938.1	488	0.432	-	4.366	34
BAM5.1_*T.cacao*_XP_007029518.1	500	0.432	-	2.071	2
BAM5.2_*A.ipaensis*_Araip.YCB0N	611	0.435	-	3.826	53
BAM5.2_*B.vulgaris*_XP_010689279.1	492	0.433	-	2.505	12
BAM5.2_*C.arietinum*_XP_004513548.1	496	0.446	-	3.365	25
BAM5.2_*E.guttatus*_XP_012852440.1	456	0.531	Y	1.283	24
BAM5.2_*G.max*_XP_003540325.2	601	0.442	-	4.941	73
BAM5.2_*G.raimondii*_XP_012479806.1	600	0.446	-	3.396	6
BAM5.2_*H.vulgare*_AAX37358.1	505	0.434	-	3.815	6
BAM5.2_*M.acuminata*_XP_009420599.1	505	0.427	-	6.204	5
BAM5.2_*M.trunculata*_XP_003597045.2	590	0.465	-	3.339	45
BAM5/6.2_*N.nucifera*_XP_010267811.1	519	0.44	-	1.914	14
BAM5.2_*O.brachyantha*_XP_006658660.1	478	0.564	Y	6.592	64
BAM5.2_*O.sativa*_NP_001059906.1	600	0.567	Y	7.392	58
BAM5.2_*P.heterocycla*_PH01002417G0300	488	0.438	-	3.815	6
BAM5.2_*S.italica*_XP_004957937.1	587	0.57	Y	6.352	35
BAM5.2_*T.cacao*_XP_007043355.1	410	0.443	-	4.354	68
BAM5.3_*B.vulgaris*_XP_010695752.1	492	0.436	-	2.505	12
BAM5.3_*G.max*_XP_003541934.2	592	0.441	-	1.594	38

BAM5.3_*H.vulgare*_BAK00030.1	603	0.548	Y	5.226	67
BAM5.3_*P.heterocycla*_PH01002346G0350	602	0.541	Y	6.878	30
BAM5_*A.corulea*_Aquca_005_00422	589	0.452	-	9.552	60
BAM5_*A.duranensis*_Aradu.A6XWX.1	527	0.436	-	2.403	14
BAM5_*C.canephora*_CDP20214.1	491	0.455	-	1.577	22
BAM5_*C.clementia*_XP_006447463.1	519	0.431	-	-1.856	2
BAM5_*C.lantus*_Cla007699	578	0.47	-	6.823	11
BAM5_*C.macrocarpa*_PNZO2024341	497	0.43	-	-2.009	18
BAM5_*C.melo*_XP_008455397.1	583	0.464	-	1.017	68
BAM5_*C.sativus*_XP_011658712.1	583	0.449	-	0.007	70
BAM5_*C.sinensis*_AHG94609.1	593	0.448	-	3.132	20
BAM5_*E.grandis*_XP_010051211.1	531	0.429	-	-0.596	81
BAM5_*M.domestica*_XP_008379598.1	598	0.513	Y	4.116	36
BAM5_*M.notabilis*_XP_010109553.1	511	0.434	-	-0.283	2
BAM5_*N.benthamiana*_Nbv5tr6213155	614	0.499	-	4.103	67
BAM5_*P.mume*_XP_008239169.1	515	0.432	-	-3.304	6
BAM5_*P.persica*_XP_007215122.1	516	0.436	-	-2.962	6
BAM5_*P.radiata*_DZQM2008648	478	0.433	-	0.568	33
BAM5_*P.taeda*_PITA_000048102RA	549	0.425	-	-2.337	19
BAM5_*P.vulgaris*_XP_007149944.1	587	0.44	-	2.451	26
BAM5_*S.bicolor*_XP_002460819.1	604	0.57	Y	4.627	51
BAM5_*S.polyrhiza*_Spipo7G0011700	401	0.487	-	7.017	15
BAM5_*S.purpurea*_SapurV1A.0312s0210	582	0.449	-	8.030	55
BAM5_*A.lyrata*_XP_002868220.1	499	0.433	-	1.136	26
BAM5_*A.thaliana*_NP_567460.1	420	0.435	-	1.783	26
BAM5_*A.trichopoda*_XP_006837006.2	717	0.479	-	5.505	49
BAM5_*E.guineesis*_XP_010931493.1	519	0.44	-	5.511	30
BAM5_*E.salsuginea*_XP_006414555.1	498	0.436	-	1.783	26
BAM5_*F.vesca*_XP_004289151.1	586	0.508	Y	1.288	31
BAM5_*J.curcas*_XP_012086395.1	518	0.433	-	3.676	28
BAM5_*P.dactylifera*_XP_008793743.1	524	0.439	-	3.727	29
BAM5_*P.vulgaris*_XP_007132589.1	497	0.443	-	4.942	45
BAM5_*R.communis*_XP_002515712.1	518	0.431	-	5.284	28
BAM5_*S.indicum*_XP_011100422.1	579	0.479	-	5.368	61
BAM5_*S.lycopersicum*_XP_004243448.1	575	0.487	-	3.975	61
BAM5_*S.tuberosum*_XP_006360578.1	578	0.508	Y	3.789	64
BAM5_*V.vinifera*_XP_002281003.2	596	0.513	Y	2.413	13
BAM5_*C.sinensis*_XP_006469732.1	519	0.431	-	-1.856	2
BAM5_*P.trichocarpa*_XP_006372990.1	583	0.434	-	-1.076	13

Table S7. Predicted BAM6 orthologs subcellular localization.

Name	Length	Score	cTP	CS-score	cTP-length
BAM6_*A.thaliana*_NP_180788.2	577	0.464	-	3.771	52
BAM6_*A.lyrata*_XP_002881219.1	577	0.457	-	0.162	5
BAM6.1_*C.sativa*_XP_010510054.1	577	0.516	Y	4.536	49
BAM6.2_*C.sativa*_XP_010414016.1	570	0.534	Y	4.861	45
BAM6.3_*C.sativa*_XP_010469618.1	577	0.509	Y	2.629	50
BAM6_*C.rubella*_XP_006293904.1	576	0.492	-	2.629	49
BAM6_*E.salsuginea*_XP_006410362.1	587	0.513	Y	8.671	27
BAM6_*B.rapa*_XP_009143965.1	581	0.534	Y	4.371	51
BAM6.1_*B.napus*_XP_013748682.1	581	0.529	Y	4.371	51
BAM6.2_*B.napus*_XP_013675826.1	582	0.492	-	4.727	52
BAM6_*T.hassleriana*_XP_010522319.1	592	0.51	Y	6.190	59

Table S8. Predicted BAM7 orthologs subcellular localization.

Name	NLS (0.6 cutoff)	NLS-sequence
BAM7.1_*B.napus*_XP_009143179.1	Y	63 - RRSRPVEEKERTKLRERHRRAI - 84
BAM7.1_*C.sativa*_XP_010508098.1	Y	66 - SRRSRPLEEKERTKLRERHRRAIT - 89
BAM7.1_*G.max*_XP_003534564.1	Y	83 - RRSRPLEEKERTKLRERRRRAITA - 106
BAM7.1_*G.raimondii*_XP_012475200.1	Y	79 - ARRSRPLEEKERTKLRERHRRAI - 101
BAM7.1_*N.nucifera*_XP_010241901.1	Y	77 - RPKEEKERTKLRERHRRS - 94
BAM7.1_*P.heterocycla*_PH01001239G0650	Y	51 - RRSRAREEKERTKLRERQRRAI - 72
BAM7.1_*P.trichocarpa*_XP_002320793.2	Y	65 - ERTKLRERHRR - 75
BAM7.2_*B.napus*_XP_013748222.1	Y	63 - RRSRPVEEKERTKLRERHRRAI - 84
BAM7.2_*C.sativa*_XP_010506568.1	Y	66 - SRRSRPLEEKERTKLRERHRRAIT - 89
BAM7.2_D.carota_KZM88115.1	-	-
BAM7.2_*G.max*_XP_003552392.1	Y	83 - RRSRPLEEKERTKLRERRRRAITA - 106
BAM7.2_*N.nucifera*_XP_010255372.1	Y	74 - PRRCRPKEEKERTKLRERHRRAIT - 97
BAM7.2_*P.heterocycla*_PH01002350G0210	Y	60 - GGRRSRAREEKERTKLRERQRRA - 82
BAM7.2_*P.trichocarpa*_XP_002302585.2	Y	77 - ARRSRPLEEKERTKLRERHRRAI - 99
BAM7.2_*S.purpurea*_SapurV1A.0046s0400	Y	76 - RRSRPLEEKERTKLRERHRRAI - 97
BAM7.3_*C.sativa*_XP_010518231.1	Y	66 - SRRSRPLEEKERTKLRERHRRAIT - 89
BAM7_*A.corulea*_Aquca_013_00313	Y	94 - RRCRPREEKERTKLRERHRRAI - 115
BAM7_*A.duranensis*_Aradu.R0WSE.1	Y	37 - KERTKLRERRRRAITA - 52
BAM7_*A.ipaensis*_Araip.5ZJ5X.1	Y	90 - KERTKLRERRRRAITA - 105
BAM7_*A.lyrata*_XP_002882038.1	Y	66 - SRRSRPLEEKERTKLRERHRRAIT - 89
BAM7_*A.thaliana*_NP_182112.2	Y	67 - SRRSRPLEEKERTKLRERHRRAIT - 90
BAM7_*A.trichopoda*_XP_006827627.2	Y	70 - RRRCRPKEEKERTKLRERHRRAIT - 93
BAM7_*B.rapa*_XP_009143179.1	Y	63 - RRSRPVEEKERTKLRERHRRAI - 84
BAM7_*B.vulgaris*_XP_010670436.1	Y	76 - RRSRPLEEKERTKLRERHRRAITARILAGLRR - 107
BAM7_*C.annum*_Capana08g000917	-	-
BAM7_*C.canephora*_CDP08819.1	-	-
BAM7_*C.cardunculus*_KVH89110.1	-	-
BAM7_*C.clementia*_XP_006445048.1	-	-
BAM7_*C.lantus*_Cla001224	Y	84 - KERTKLRERHRRA - 96
BAM7_*C.melo*_XP_008458240.1	Y	84 - KERTKLRERHRRA - 96
BAM7_*C.papaya*_evm.model.supercontig_18.252	Y	88 - KERTKLRERHRRA - 100
BAM7_*C.sativus*_XP_011656338.1	Y	84 - KERTKLRERHRRA - 96
BAM7_*C.sinensis*_AKQ62959.1	Y	83 - EKERKKIRERQRRAV - 97
BAM7_*E.grandis*_XP_010055132.1	Y	75 - GARRSRPLEEKERTKLRERHRRAI - 98
BAM7_*E.guineesis*_XP_010909265.1	Y	83 - RRPRAKEEKERTKMRERHRRAI - 104

BAM7_*E.guttatus*_XP_012840197.1	-	-
BAM7_*E.salsuginea*_XP_006397762.1	Y	66 - SRRSRPLEEKERTKLRERHRRAIT - 89
BAM7_*F.vesca*_XP_004306787.1	Y	92 - TKLRERQRR - 100
BAM7_*G.racemosa*_QIKZ2024994	Y	89 - RER - 91
BAM7_*H.vulgare*_BAJ96466.1	Y	40 - RPPERRRGRGREEKERTKARERRRRAVTGRILAGLRRH - 77
BAM7_*I.vomitoria*_ASMV2024789	-	-
BAM7_*J.curcas*_XP_012083397.1	Y	77 - GARRSRPLEEKERTKLRERHRRAI - 100
BAM7_*M.acuminata*_XP_009398621.1	Y	80 - SRRSRPAEEKERTKLRERHRRAITG - 104
BAM7_*M.domestica*_XP_008338860.1	Y	93 - RTKLRERQR - 101
BAM7_*M.esculenta*_cassava4.1_002728m	Y	78 - ARRSRPLEEKERTKLRERHRRAI - 100
BAM7_*M.notabilis*_XP_010105937.1	Y	90 - KERTKLRERHRRA - 102
BAM7_*M.trunculata*_XP_013449334.1	Y	80 - NRRSRPVEEKERTKLRERRRRAITA - 104
BAM7_*N.benthamiana*_Nbv5tr6219619	Y	85 - RER - 87
BAM7_*P.dactylifera*_XP_008777296.1	Y	83 - RRPRPKEEKERTKMRERHRRAIT - 105
BAM7_*P.mume*_XP_008232902.1	-	-
BAM7_*P.persica*_XP_007220223.1	-	-
BAM7_*P.vulgaris*_XP_007139874.1	Y	81 - RRSRPVEEKERTKLRERRRRAITA - 104
BAM7_*R.communis*_XP_002511857.1	Y	83 - RSRPLEEKERTKLRERHRRAI - 103
BAM7_*S.indicum*_XP_011093139.1	Y	97 - KLRERQR - 103
BAM7_*S.lycopersicum*_XP_004229887.1	Y	86 - RERQ - 89
BAM7_*S.polyrhiza*_Spipo1G0030700	Y	99 - RCRPKEEKERTKLRERHRRAI - 119
BAM7_*S.tuberosum*_XP_006339564.1	Y	85 - LRERQR - 90
BAM7_*T.cacao*_XP_007051814.1	Y	78 - ARRSRPLEEKERTKLRERHRRAI - 100
BAM7_T.hassleriana_XP_010523740.1	Y	66 - SRRSRPLEEKERTKLRERHRRAIT - 89

Table S9. Predicted BAM8 orthologs subcellular localization.

Name	NLS (0.6 cutoff)	NLS-sequence
BAM8.1_*B.napus*_XP_013721726.1	Y	73 - GGGGGKGKREREKEKERTKLRERHRRA - 99
BAM8.1_*C.sativa*_XP_010441757.1	Y	75 - GERGKGKREREKEKERTKLRERHRRA - 100
BAM8.2_*B.napus*_XP_013656407.1	Y	72 - GGGGKGKREREKEKERTKLRERHRRA - 97
BAM8.2_*C.sativa*_XP_010481609.1	Y	59 - GGGGKGKREREKEKERTKLRERHRRA - 84
BAM8.2_*C.sativa*_XP_010494622.1	Y	76 - GGERGKGKREREKEKERTKLRERHRRA - 102
BAM8.2_*S.indicum*_XP_011082155.1	Y	66 - KSRKEREKEKERTKLRERHRRA - 87
BAM8_*A.lyrata*_XP_002865252.1	Y	76 - GGGGKGKREREKEKERTKLRERHRRA - 101
BAM8_*A.thaliana*_NP_199343.1	Y	79 - GGGGGKGKREREKEKERTKLRERHRRA - 105
BAM8_*A.trichopoda*_XP_011624011.1	Y	73 - KGRKEREKEKERTKLRERHRRA - 94
BAM8_*B.rapa*_XP_009128781.1	Y	72 - GGGKGKREREKEKERTKLRERHRRA - 96
BAM8_*B.vulgaris*_XP_010676684.1	Y	67 - GKGRREREKEKERTKLRERHRRA - 89
BAM8_*C.annum*_Capana01g004209	Y	66 - KSRKEREKEKERTKLRERHRRA - 87
BAM8_*C.arietinum*_XP_004512346.1	Y	46 - GKGKKEREKEKERTKLRERHRRA - 68
BAM8_*C.cardunculus*_KVH95140.1	Y	57 - SRNEREKEKERTKLRERHRR - 76
BAM8_*C.clementia*_XP_006432891.1	Y	65 - GKGKKEREKEKERTKLRERHRRA - 87
BAM8_*C.lantus*_Cla005462	Y	61 - GKAKREREKEKERTKLRERHRRA - 83
BAM8_*C.melo*_XP_008451866.1	Y	60 - GKAKREREKEKERTKLRERHRRA - 82
BAM8_*C.rubella*_XP_006279583.1	Y	89 - GGGGKGKREREKEKERTKLRERHRRA - 114
BAM8_*C.sativus*_XP_011653241.1	Y	61 - GKAKREREKEKERTKLRERHRRA - 83
BAM8_*C.sinensis*_AKQ62960.1	Y	48 - RRPRGFAASSSSGVAKGKKEREKEKERTKLRERHRRA - 84
BAM8_*C.sinensis*_XP_006494107.1	Y	65 - GKGKKEREKEKERTKLRERHRRA - 87
BAM8_*E.grandis*_XP_010054915.1	Y	60 - PGKGKREREKEKERTKLRERHRRA - 83
BAM8_*E.guttatus*_XP_012837341.1	Y	68 - KSRKEREKEKERTKLRERHRRA - 89
BAM8_*E.salsuginea*_XP_006398198.1	Y	77 - GGGGGKGKREREKEKERTKLRERHRRA - 103
BAM8_*F.vesca*_XP_011465289.1	Y	54 - ISPSTKGRREREKEKERTKLRERLRR - 79
BAM8_*G.max*_XP_003516502.1	Y	53 - GGKGKKEREKEKERTKLRERHRRA - 76
BAM8_*G.racemosa*_QIKZ2029211	Y	70 - KGRKEREKEKERTKLRERHRRA - 91
BAM8_*G.raimondii*_XP_012439178.1	Y	46 - GKGKREREKEKERTKLRERHRRA - 68
BAM8_*J.curcas*_XP_012083880.1	Y	69 - GKGKREREKEKERTKLRERHRRA - 91
BAM8_*M.acuminata*_XP_009384530.1	Y	73 - AVAKGRKEREKEKERTKLRERHRRA - 97
BAM8_*M.domestica*_XP_008373437.1	Y	72 - KGKREREKEKERTKLRERLRR - 92
BAM8_*M.notabilis*_XP_010105162.1	Y	62 - SKGGKREREREKEKERTKLRERHRRA - 87
BAM8_*M.trunculata*_XP_003612541.1	Y	47 - GKGKKEREKEKERTKLRERHRRA - 69
BAM8_*N.benthamiana*_Nbv5tr6214148	Y	44 - AGATNKNRKEREKEKERTKLRERHRRA - 70

BAM8_*N.nucifera*_XP_010275178.1	Y	46 - GGKGKKEKRTKLRERHRRA - 64
BAM8_*P.dactylifera*_XP_008808792.1	Y	34 - AAAAGGSGKCRKEREKEKERTKLRERHRR - 62
BAM8_*P.mume*_XP_008238070.1	Y	91 - NKGKREREREKERTKLRERLRR - 112
BAM8_*P.persica*_XP_007210828.1	Y	69 - KGKREREREKERTKLRERLRR - 89
BAM8_*P.trichocarpa*_XP_002304400.1	Y	73 - GKGKREREKEKERTKLRERHRRA - 95
BAM8_*P.vulgaris*_XP_007158095.1	Y	55 - AKGKKEREKEKERTKLRERHRRA - 77
BAM8_*R.communis*_XP_002519919.1	Y	71 - RGKREREKEKERTKLRERHRRA - 92
BAM8_*S.bicolor*_XP_002451472.1	Y	16 - PPQRRPRGFASTAGGSPRRRGEREREREKERTKLRERHRRA - 56
BAM8_*S.lycopersicum*_XP_004244442.1	Y	60 - KSRKEREKEKERTKLRERHRRA - 81
BAM8_*S.purpurea*_SapurV1A.0961s0160	Y	76 - GKGKREREKEKERTKLRERHRRA - 98
BAM8_*S.tuberosum*_XP_006361593.1	Y	11 - KSRKEREKEKERTKLRERHRRA - 32
BAM8_*T.cacao*_XP_007040897.1	Y	60 - GKGKREREKEKERTKLRERHRRA - 82
BAM8_*T.hassleriana*_XP_010529274.1	Y	65 - GSGGGGAKGKREREKEKERTKLRERHRRA - 93
BAM8_*V.vinifera*_XP_002270680.1	Y	59 - GGGGGGKGKKEREKEKERTKLRERHRRA - 86
BAM8_*Z.mays*_XP_008679986.1	Y	19 - RRPRGFASAPAPAAGASPRRRGVQEREREREKERTKLRERHRRA - 62

Table S10. Predicted BAM9 orthologs subcellular localization.

Name	Length	Score	cTP	CS-score	cTP-length
BAM9.1_*A.lyrata*_XP_002871828.1	534	0.445	-	0.548	15
BAM9.1_*B.distachyon*_XP_010229570.1	537	0.567	Y	6.588	64
BAM9.1_*B.napus*_XP_013730262.1	530	0.473	-	10.228	59
BAM9.1_*B.rapa*_XP_009131684.1	530	0.463	-	10.228	59
BAM9.1_*C.sativa*_XP_010492888.1	533	0.484	-	9.325	55
BAM9.1_*G.max*_XP_003542915.1	536	0.473	-	2.186	14
BAM9.1_*G.raimondii*_XP_012454525.1	536	0.49	-	-0.54	64
BAM9.1_*M.acuminata*_XP_009399963.1	532	0.557	Y	6.836	67
BAM9.1_*M.domestica*_XP_008390741.1	529	0.471	-	7.893	62
BAM9.1_*N.nucifera*_XP_010241169.	541	0.464	-	8.481	73
BAM9.1_*O.sativa*_NP_001060573.1	523	0.468	-	6.389	16
BAM9.1_*P.heterocycla*_PH01003421G0090	488	0.44	-	7.157	35
BAM9.1_*S.bicolor*_XP_002463351.1	531	0.548	Y	6.25	51
BAM9.1_*S.indicum*_XP_011090854.1	539	0.542	Y	8.922	66
BAM9.1_*S.italica*_XP_004958614.1	524	0.537	Y	3.51	47
BAM9.1_*S.polyrhiza*_Spipo3G0042000	549	0.546	Y	10.142	63
BAM9.1_*Z.mays*_NP_001170007.1	531	0.56	Y	4.437	51
BAM9.2_*A.lyrata*_XP_002884575.1	453	0.441	-	12.423	62
BAM9.2_*B.distachyon*_XP_003561633.1	518	0.507	Y	4.715	20
BAM9.2_*B.napus*_XP_013683187.1	536	0.451	-	10.936	63
BAM9.2_*B.rapa*_XP_009120950.1	537	0.451	-	10.936	64
BAM9.2_*C.sativa*_XP_010454120.1	533	0.485	-	10.228	62
BAM9.2_*G.max*_NP_001236364.1	536	0.472	-	2.186	14
BAM9.2_*G.raimondii*_XP_012482083.1	535	0.54	Y	7.604	59
BAM9.2_*M.acuminata*_XP_009391567.1	531	0.488	-	6.023	63
BAM9.2_*M.domestica*_XP_008340845.1	530	0.511	Y	8.901	63
BAM9.2_*N.nucifera*_XP_010245368.1	543	0.515	Y	2.044	52
BAM9.2_*O.brachyantha*_XP_006651377.1	299	0.495	-	4.264	11
BAM9.2_*O.sativa*_NP_001050116.2	524	0.504	Y	6.54	11
BAM9.2_*P.heterocycla*_PH01001710G0200	523	0.517	Y	3.19	6
BAM9.2_*S.bicolor*_XP_002467860.1	529	0.519	Y	7.081	51
BAM9.2_*S.indicum*_XP_011071485.1	539	0.542	Y	8.804	64
BAM9.2_*S.italica*_XP_004984382.1	521	0.488	-	8.519	49
BAM9.2_*S.polyrhiza*_Spipo22G0010700	556	0.504	Y	11.858	68
BAM9.2_*Z.mays*_NP_001151271.2	537	0.54	Y	8.352	55
BAM9.3_*C.sativa*_XP_010420647.1	533	0.474	-	9.325	55
BAM9_*A.corulea*_Aquca_003_00854	532	0.484	-	2.118	60
BAM9_*A.thaliana*_NP_197368.1	536	0.441	-	9.325	55
BAM9_*A.trichopoda*_XP_006855410.1	524	0.431	-	2.733	9

BAM9_*B.vulgaris*_XP_010666684.1	539	0.476	-	6.595	66
BAM9_*C.annum*_Capana01g003107	534	0.503	Y	1.067	60
BAM9_*C.arietinum*_XP_004486065.1	536	0.514	Y	1.398	33
BAM9_*C.canephora*_CDO98919.1	540	0.478	-	8.253	66
BAM9_*C.cardunculus*_KVH91414.1	819	0.467	-	3.725	55
BAM9_*C.clementia*_XP_006419671.1	543	0.486	-	8.526	26
BAM9_*C.lantus*_Cla009332	532	0.513	Y	5.572	55
BAM9_*C.melo*_XP_008458491.1	533	0.521	Y	3.782	55
BAM9_*C.papaya*_evm.model.supercontig_16.112	546	0.471	-	10.076	72
BAM9_*C.rubella*_XP_006287474.1	532	0.479	-	7.803	63
BAM9_*C.sativus*_XP_004153140.1	532	0.51	Y	5.572	55
BAM9_*C.sinensis*_XP_006489160.1	543	0.483	-	8.526	26
BAM9_*D.carota*_KZM87479.1	532	0.483	-	5.188	57
BAM9_*E.grandis*_XP_010024561.1	532	0.511	Y	2.64	55
BAM9_*E.guineesis*_XP_010938702.1	530	0.485	-	9.64	60
BAM9_*E.guttatus*_XP_012827989.1	374	0.55	Y	5.426	67
BAM9_*E.salsuginea*_XP_006400419.1	533	0.449	-	9.325	55
BAM9_*F.vesca*_XP_004296793.1	530	0.479	-	2.908	54
BAM9_*G.racemosa*_QIKZ2016136	526	0.573	Y	6.215	53
BAM9_*H.vulgare*_BAK03717.1	526	0.516	Y	0.841	46
BAM9_*I.vomitoria*_ASMV2109307	540	0.505	Y	9.347	65
BAM9_*J.curcas*_XP_012069407.1	532	0.499	-	6.627	69
BAM9_*M.esculenta*_cassava4.1_005239m	535	0.506	Y	0.987	72
BAM9_*M.notabilis*_XP_010105020.1	535	0.49	-	8.398	64
BAM9_*M.trunculata*_XP_003594004.1	535	0.448	-	6.297	64
BAM9_*N.benthamiana*_Nbv5tr6214300	540	0.482	-	3.038	64
BAM9_*P.dactylifera*_XP_008796202.1	524	0.471	-	7.987	60
BAM9_*P.mume*_XP_008223100.1	530	0.485	-	8.901	62
BAM9_*P.persica*_XP_007222488.1	529	0.489	-	8.901	62
BAM9_*P.trichocarpa*_XP_002312750.2	535	0.519	Y	3.265	60
BAM9_*P.vulgaris*_XP_007147864.1	532	0.493	-	5.875	64
BAM9_*R.communis*_XP_002516865.1	545	0.516	Y	6.222	81
BAM9_*S.lycopersicum*_NP_001234052.1	535	0.56	Y	3.038	65
BAM9_*S.purpurea*_SapurV1A.0022s0350	535	0.566	Y	7.113	60
BAM9_*S.tuberosum*_XP_006342739.1	535	0.558	Y	3.038	65
BAM9_*T.cacao*_XP_007035476.1	537	0.491	-	2.061	71
BAM9_*T.hassleriana*_XP_010558493.1	536	0.46	-	12.7	62
BAM9_*V.vinifera*_XP_002276777.1	541	0.49	-	6.827	65

Table S11. Predicted BAM10 orthologs subcellular localization.

Name	Length	Score	cTP	CS-score	cTP-length
BAM10.1_*G.max*_XP_003532447.1	553	0.58	Y	2.400	40
BAM10.1_*M.domestica*_XP_008390323.1	559	0.57	Y	1.909	59
BAM10.1_*Z.mays*_NP_001130896.1	539	0.59	Y	6.177	47
BAM10.2_*G.max*_XP_003525331.1	557	0.57	Y	0.527	49
BAM10.2_*M.domestica*_XP_008337562.1	559	0.57	Y	6.923	59
BAM10.2_*Z.mays*_NP_001132696.1	537	0.59	Y	1.833	45
BAM10_*A.duranensis*_Aradu.FYP9T.1	544	0.58	Y	2.331	59
BAM10_*A.trichopoda*_XP_006844925.1	559	0.58	Y	5.275	54
BAM10_*B.distachyon*_XP_003566188.1	556	0.58	Y	3.029	45
BAM10_*C.arietinum*_XP_004503587.1	554	0.57	Y	0.970	40
BAM10_*C.clementia*_XP_006439286.1	541	0.56	Y	3.872	99
BAM10_*C.lantus*_Cla002226	548	0.58	Y	0.448	50
BAM10_*C.melo*_XP_008460412.1	546	0.58	Y	0.448	50
BAM10_*C.sativus*_XP_004144400.1	546	0.58	Y	0.564	36
BAM10_*Camellia.sinensis*_AKQ62958.1	549	0.56	Y	5.177	27
BAM10_*Citrus.sinensis*_XP_006476339.1	536	0.56	Y	3.872	99
BAM10_*E.grandis*_XP_010055984.1	551	0.56	Y	3.066	31
BAM10_*E.guineesis*_XP_010915994.1	550	0.57	Y	1.944	66
BAM10_*E.guttatus*_XP_012842111.1	518	0.54	Y	4.132	49
BAM10_*F.vesca*_XP_004301815.1	542	0.58	Y	3.347	46
BAM10_*H.vulgare*_BAJ96156.1	547	0.57	Y	7.440	32
BAM10_*J.curcas*_XP_012086671.1	553	0.59	Y	3.709	50
BAM10_*M.acuminata*_XP_009403535.1	542	0.56	Y	1.529	44
BAM10_*M.acuminata*_XP_009409087.1	561	0.56	Y	1.997	74
BAM10_*M.notabilis*_XP_010107262.1	560	0.57	Y	1.230	19
BAM10_*M.trunculata*_XP_013447245.1	545	0.58	Y	1.909	47
BAM10_*N.benthamiana*_Nbv5tr6219815	548	0.57	Y	2.301	49
BAM10_*O.sativa*_NP_001172248.1	587	0.58	Y	2.532	66
BAM10_*P.dactylifera*_XP_008783150.1	550	0.56	Y	1.697	55
BAM10_*P.mume*_XP_008239169.1	567	0.58	Y	3.766	57
BAM10_*P.persica*_XP_007209090.1	567	0.58	Y	3.766	57
BAM10_*P.trichocarpa*_XP_002297961.1	555	0.58	Y	1.286	43
BAM10_*P.vulgaris*_XP_007160198.1	549	0.57	Y	-0.451	42
BAM10_*S.indicum*_XP_011070357.1	538	0.55	Y	4.132	51
BAM10_*S.italica*_XP_004967358.1	544	0.59	Y	2.132	42
BAM10_*S.lycopersicum*_XP_004245482.1	539	0.57	Y	4.131	47
BAM10_*S.polyrhiza*_Spipo8G0049600	568	0.58	Y	5.189	47
BAM10_*S.tuberosum*_XP_006343811.1	541	0.57	Y	4.976	47
BAM10_*T.cacao*_XP_007040595.1	627	0.57	Y	1.952	62

BAM10_*V.vinifera*_XP_010659745.1	542	0.56	Y	0.893	48
