# Supplementary material for: The evolution of functional complexity within the β-amylase gene family in land plants
Source: BMC Evol Biol. 2019 Feb 28;19:66. doi: 10.1186/s12862-019-1395-2 (PMC6394054; doi:10.1186/s12862-019-1395-2)
Supplement: Supplementary file 5 — SolycBAM10 gene is expressed in most plant tissues. (PDF 193 kb) [file 12862_2019_1395_MOESM5_ESM.pdf]

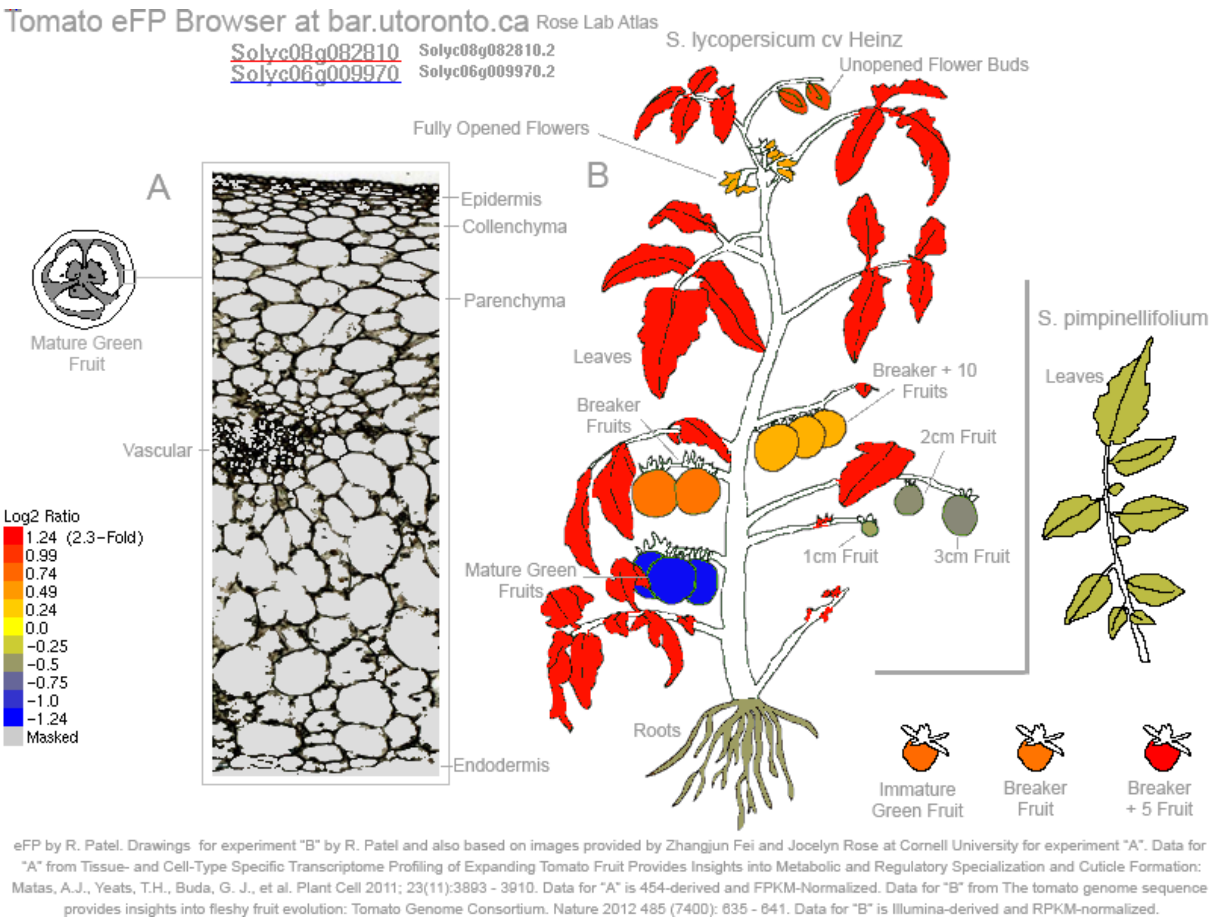

Additional file 5. *SolycBAM10* gene is expressed in most plant tissues. Comparison of expression profile of *BAM10* gene from tomato (*Solyc08g082810*) relative to the housekeeping gene *SolycElf1*α (*Solyc06g009970*). Data, including drawings, were retrieved from the public Tomato eFP browser microarray dataset “Rose Lab Atlas” (http://bar.utoronto.ca/efp_tomato/cgi-bin/efpWeb.cgi).
