# Supplementary material for: The evolution of functional complexity within the β-amylase gene family in land plants
Source: BMC Evol Biol. 2019 Feb 28;19:66. doi: 10.1186/s12862-019-1395-2 (PMC6394054; doi:10.1186/s12862-019-1395-2)
Supplement: Supplementary file 6 — Protein alignment of BAM isoforms from Arabidopsis and BAM10 from Theobroma cacao. (PDF 1188 kb) [file 12862_2019_1395_MOESM6_ESM.pdf]

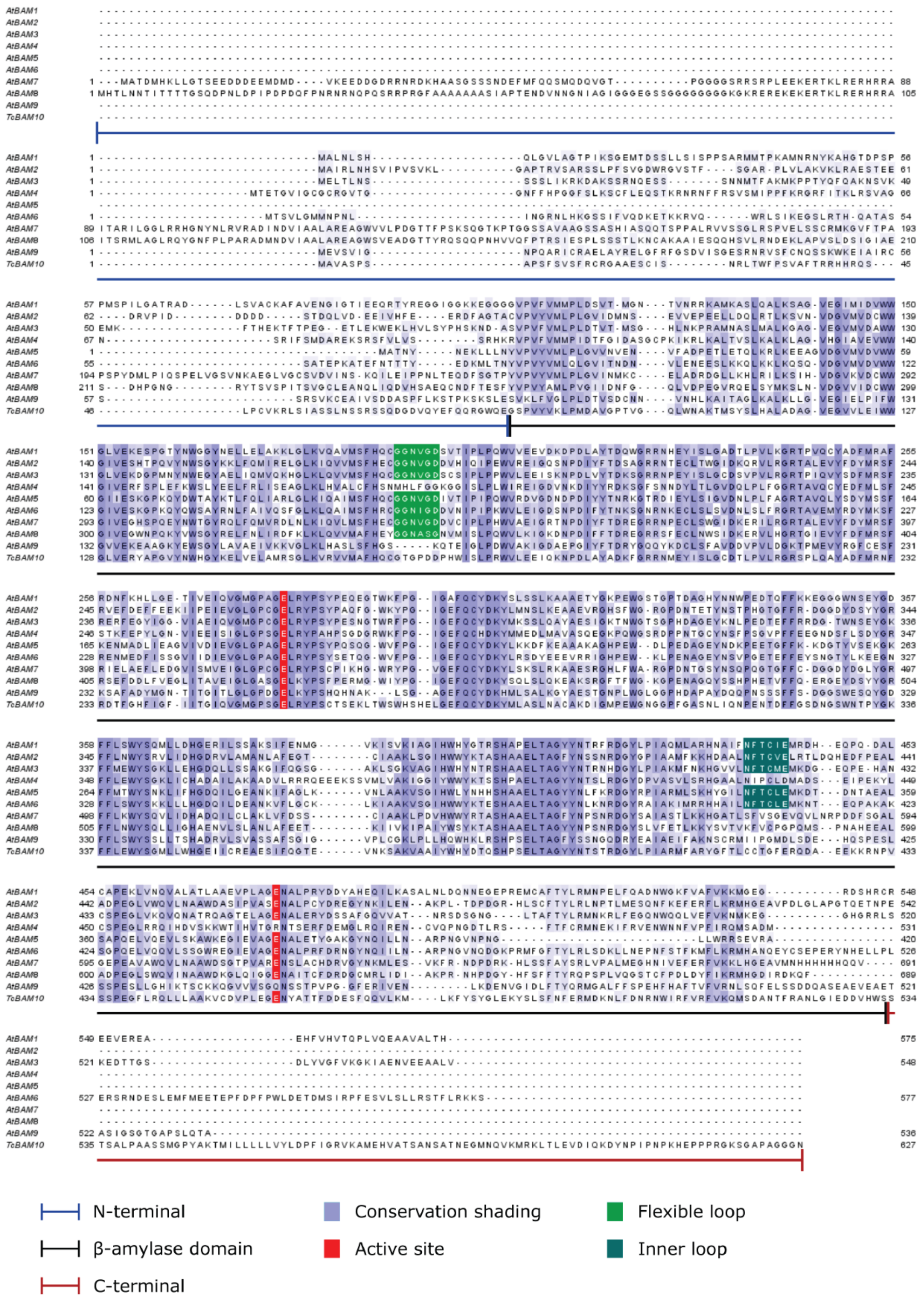

Additional file 6. Amino acid sequence comparison of BAM isoforms from Arabidopsis and *Theobroma cacao*. The alignments were generated with ClustalW2 program and then adjusted manually. Lines below the sequences display the N-terminal domain (in blue), the β-amylase domain (in black) and the C-terminal domain (in red), according to UniProt (http://www.uniprot.org/uniprot/Q9LIR6) and InterPro (https://www.ebi.ac.uk/interpro/).
