# Supplementary material for: The evolution of functional complexity within the β-amylase gene family in land plants
Source: BMC Evol Biol. 2019 Feb 28;19:66. doi: 10.1186/s12862-019-1395-2 (PMC6394054; doi:10.1186/s12862-019-1395-2)
Supplement: Supplementary file 7 — Protein alignment of BZR1-domain containing β-amylases from representative non-flowering plants. (PDF 159 kb) [file 12862_2019_1395_MOESM7_ESM.pdf]

```
BAM2_Arabidopsis thaliana         1 ------------------------------------------------------------
BAM7_Arabidopsis thaliana         1 ----MATDMHKLLGTSEEDDDEE-MDMDVKEEDDGDRRNRDKHAASGSSSNDEFMFQQ--
BAM7_Amborella trichopoda         1 MAQEMEGNGDDVVGEEEEEDEEDDMEMEVKEEEEDEEAQNQSHHARLQMQQPHHVQSS--
BAM8_Amborella trichopoda         1 ----MDYNRGVEMSVKQDLDHNNDHHLQAQIPP-----QLQVQIPS--QAQVQNQLIQTP
BAM8_Arabidopsis thaliana         1 ----MHTLNNTITTTTGSQDPNLDPIPDPDQFPNRNRNQPQSRRPRGFAAAAAAASIAPT
BZR-BAM_Abies lasiocarpa          1 ------------------------------------------------------------
BZR-BAM_Pseudolarix amabilis      1 ------------------------------------------------------------
BZR-BAM_Pseudotsuga wilsoniana    1 ------------------------------------------------------------
BZR-BAM_Picea engelmandii         1 ------------------------------------------------------------
BZR-BAM_Tsuga heterophylla        1 ------------------------------------------------------------
BZR-BAM_Wollemia nobilis          1 ------------------------------------------------------------
BZR-BAM_Agathis robusta           1 ------------------------------------------------------------
BZR-BAM_Podocarpus rubens         1 ------------------------------------------------------------
BZR-BAM_Cupressus dupreziana      1 ------------------------------------------------------------
BZR-BAM_Taiwania cryptomerides    1 ------------------------------------------------------------
BZR-BAM_Stangeria eriopus         1 ------------------------------------------------------------
BZR-BAM_Encephalartos barteri     1 ------------------------------------------------------------
BZR-BAM_Ophioglossum vulgatum     1 ------------------------------------------------------------
```

BZR1-like domain

```
BAM2_Arabidopsis thaliana         1 ------------------------------------------------------------
BAM7_Arabidopsis thaliana        54 --------------SMQDQVGTPGGGGSRRSRPLEEKERTKLRERHRRAITARILGGLRR
BAM7_Amborella trichopoda        59 --------------ELQQQGGSVEG--RRRCRPKEEKERTKLRERHRRAITARILAGLRR
BAM8_Amborella trichopoda        50 QNETPRR----LRGFAATAAGVTSLSTKGRKEREKEKERTKLRERHRRAITSRMLAGLRQ
BAM8_Arabidopsis thaliana        57 ENDVNNGNIAGIGGGEGSSGGGGGGGGKGKREREKEKERTKLRERHRRAITSRMLAGLRQ
BZR-BAM_Abies lasiocarpa          1 --------------QQVLAPAGNATVKRRIREREKEKERTKLRERQRRAVTSRILSGLRQ
BZR-BAM_Pseudolarix amabilis      1 ------------------------------REREKEKERTKLRERQRRAITSRILSGLRQ
BZR-BAM_Pseudotsuga wilsoniana    1 ----------QGNVQQVLVPAGNATVKRRIREREKEKERTKLRERQRRAITSRILSGLRQ
BZR-BAM_Picea engelmandii         1 --------------QQVLLPAGNATVKRRIREREKEKERTKLRERQRRAITSRILSGLRQ
BZR-BAM_Tsuga heterophylla        1 ----------QGNVQQVIVAAGNATVKRRIREREKEKERTKLRERQRRAVTSRILSGLRQ
BZR-BAM_Wollemia nobilis          1 ---------------------------RRIREREKEKERTKLRERQRRAITSRILSGLRQ
BZR-BAM_Agathis robusta           1 ------------------------------REREKEKERTKLRERQRRAITSRILSGLRQ
BZR-BAM_Podocarpus rubens         1 ---------------------------RRVREREKEKERTKLRERQRRAITSRILSGLRQ
BZR-BAM_Cupressus dupreziana      1 ------------------------------REREKERERTKLRERQRRAITSRILSGLRQ
BZR-BAM_Taiwania cryptomerides    1 -----------------QRQGHPVTAKRRIREREKEKERTKLRERQRRAITSRILSGLRQ
BZR-BAM_Stangeria eriopus         1 ------------------------------REREKEKERTKLRERQRRAVTSRILSGLRQ
BZR-BAM_Encephalartos barteri     1 ------------------------------REREKEKERTKLRERQRRAVTSRILSGLRQ
BZR-BAM_Ophioglossum vulgatum     1 -----------------------------EKDREKEKERTKLRERQRRAITSKILTGLRR
```

BZR1-like domain

```
BAM2_Arabidopsis thaliana         1 ---MAIRLNHSVIPVSVKLG-------------------------APTRVSARSSLPFSV
BAM7_Arabidopsis thaliana       100 HGNYNLRVRADINDVIAALAREAGWVVLPDGTTFPSKSQGTKPTGGSSAVAAGSSASHIA
BAM7_Amborella trichopoda       103 HGNYNLRVRADINDVIAALAREAGWMVLPDGTTFPSRPHNAR--TAATPSTPTTSSPLPA
BAM8_Amborella trichopoda       106 YGNFTLPARADMNDVLAALAREAGWTVEPDGTTYRTS---------PATLTPFP-IRAVE
BAM8_Arabidopsis thaliana       117 YGNFPLPARADMNDVIAALAREAGWSVEADGTTYRQSQ-------QPNHVVQFP-TRSIE
BZR-BAM_Abies lasiocarpa         47 YGNYTLPVRADINDVIAALAREAGWTVDSDGTTYRSQ------TPYPLKVAAPSSTSLAG
BZR-BAM_Pseudolarix amabilis     31 YGNYTLPVRADINDVIAALAREAGWTVDSDGTTYRSQT----HTPYPLKVAAPSSTSLAG
BZR-BAM_Pseudotsuga wilsoniana   51 YGNYTLPARADINDVIAALAREAGWTVDSDGTTYRSQA----HTPYPVKVAALSSTSLAG
BZR-BAM_Picea engelmandii        47 YGNYTLPVRADINDVIAALAREAGWTVDPDGTTYRSQA----HTPYPVKVAALSSTSLAG
BZR-BAM_Tsuga heterophylla       51 YGNYTLPVRADINDVIAALAREAGWTVDSDGTTYRSQA----HTSYPLKVAAPSSTSLAG
BZR-BAM_Wollemia nobilis         34 YGNYNLPVRADINDVIAALAREAGWTVEPDGTTYRLHSHS--HTPYPPKVAALSSTSLVG
BZR-BAM_Agathis robusta          31 YGNYNLPVRADINDVIAALAREAGWTVEPDGTTYRLHSPS--RTPYPPKVAALSSTSLVG
BZR-BAM_Podocarpus rubens        34 YGNYNLPVRADINDVLAAVAREAGWTVEADGTTYKLHT------PYPSKVAALSSVSLAG
BZR-BAM_Cupressus dupreziana     31 YGNYSLPVRADINDVIAALAREAGWTVDSDGTTYRPLSLS--HIAYPPKLAALSSTSHVG
BZR-BAM_Taiwania cryptomerides   44 YGNYSLPVRADINDVIAALAREAGWTVDPDGTTYRPLSLS--HIPYPPKVAALSSTSLVG
BZR-BAM_Stangeria eriopus        31 YGNYSLPVRADINDVIAALAREAGWTVESDGTTYRSHS----HTPYPPRVAPLSSTSLIG
BZR-BAM_Encephalartos barteri    31 FGNYSLPVRADINDVIAALAREAGWTVESDGTTYRSHS----HTPYPPRVAALSSTSLVG
BZR-BAM_Ophioglossum vulgatum    32 YGNYNLPARADINDVIKALAREAGWNVEADGTTYRSRVR---YPPAPWGNFAGGKEILPS
```

```
BAM2_Arabidopsis thaliana           33 GDWR------GVSTFSGAR------------------------PLVLAKVKLRAESTEED
BAM7_Arabidopsis thaliana          160 SQQTSPP--ALRVVSSGLRSPVELSSCRMKGVFTPAPSPYD--MLPIQSPELVGSVNKAE
BAM7_Amborella trichopoda          161 SQLPPAALRGGSATGSGFRNSVEYRPCRLKGVFVPT-SLAS--PRIVANMGDRGDKNDGS
BAM8_Amborella trichopoda          156 SPLSATS---MKNCSMKSSLDFPTSALRIEDSLSPTSPDSG---LVTEKDQK-NDKYRNP
BAM8_Arabidopsis thaliana          169 SPLSSST---LKNCAKAAIESQQHSVLRNDEKLAPVSLDSI---GIAESDHPGNGRYTSV
BZR-BAM_Abies lasiocarpa           101 SPVSSAS---LRSTLVKNPTDHRLSGVRLRDGFTQKSPGST---ILGERGPKKEKIFN--
BZR-BAM_Pseudolarix amabilis        87 SPVSSAS---LRSTLVKNPTDYRPSAVRLRDSFTLKSPGST---ILGEKGPKKEKIFN--
BZR-BAM_Pseudotsuga wilsoniana     107 SPGSSAS---LRSTLVKNPIDHRPSGVRLREGFTLKSPGST---MLGEKGPKKEKIYN--
BZR-BAM_Picea engelmandii          103 SPGSSAS---LRSTLVKNPIDHRPSGVRLRDGFTLKSPGST---MLGEKGPKKEKIYN--
BZR-BAM_Tsuga heterophylla         107 SPVSSAS---LRSTLVKNPTDYRPSGVRLRDGFTLKSPGST---ILGEKGPKKEKIFN--
BZR-BAM_Wollemia nobilis            92 S-LSSAS---LRSTLVKNSIDFRPSGLRLKDRFTVKSPVSA---IYAEKGQKKGKTIA--
BZR-BAM_Agathis robusta             89 S-LSSAS---LRSTLVKNSIDFRPSGLRLKDSFTVKSPFSA---IYAEKGQKKGKTIA--
BZR-BAM_Podocarpus rubens           88 TTISSAS---FRSALVKNSMESRPSGLRLKENYKVKTPVSA---ICGDKSQKKEKITN--
BZR-BAM_Cupressus dupreziana        89 SPVSSVS---LRSTLVKHSIDFRPSALRLKDSFTVKSPVSA---MNRDKVQKKEKTIN--
BZR-BAM_Taiwania cryptomerides     102 SPVSSAS---LRSTLVKNSIDFRPAALRLKDSFTVKSPVSA---MYGEKGQKKEKTIN--
BZR-BAM_Stangeria eriopus           87 SPVSATS---LRSTLVKTSVDFRPSGFRLKDNFAPKSPVSA---LFGEKGQMKEKTTS--
BZR-BAM_Encephalartos barteri       87 SPVSATS---LRSTLVKTSVDFRPSGFRLKDNFAPKSPVSA---LFGEK-----------
BZR-BAM_Ophioglossum vulgatum       89 QLSSSAS---VFAPSINESDVKPAEPASMKESLTPKDQATFSQKVVWEPCLANSEGVG--
```

Start of the β-amylase domain

↓

```
BAM2_Arabidopsis thaliana           63 RVPIDDDDDSTDQLVDEEIVHFEERDFAGTACVPVYVMLPLGVIDMNSEVVEPEELLDQL
BAM7_Arabidopsis thaliana          216 GLVGCSVDVINSKQILEIPPNLTEQDFSGTPYVPVYVMLPLGVINMKCELADRDGLLKHL
BAM7_Amborella trichopoda          218 PLISSCMETCDDKQVLDVSGRLPERNFAGTPYVPVYVMLPLGVINMKCEVVDLDGLVKHL
BAM8_Amborella trichopoda          209 SPIDSSDSLDADQHMRDVRPGDP-DDFSETPYIPVYVMLPLGIINNFCQLVDPEAVCRDL
BAM8_Arabidopsis thaliana          223 SPITSVGCLEANQLIQDVHSAEQCNDFTESFYVPVYAMLPVGIIDNFGQLVDPEGVRQEL
BZR-BAM_Abies lasiocarpa           153 VTVDNSVELMDEEQVQEFLPKEPERDFSGTPDVPVYVMLPLGIINDRCQLVDPEGLRKDL
BZR-BAM_Pseudolarix amabilis       139 VTIDNSLELMEEEQVQEFLPKEPERDFAGTPDVPVYVMLPLGIINDRCQLVDPEGLRKDL
BZR-BAM_Pseudotsuga wilsoniana     159 VTIDNSLELME---VQEFLPKEPERDFSGTPDVPVYVMLPLGIINDRCQLVDPEGLRKDL
BZR-BAM_Picea engelmandii          155 VAIDNSLELMEEEQVQEFLPKEPERDFSGTPDVPVYVMLPIGIINDRCQLVDPESLRKDL
BZR-BAM_Tsuga heterophylla         159 LTIDNSLELME---VQEFLPKVPERDFSGTPDVPVYVMLPLGIINDRCQLVDPEGLRKDL
BZR-BAM_Wollemia nobilis           143 LSIDNSLELMEEEQVQEFIPKEPERDFAGTPYVPVYVMLPLGIINDRCQLVDPEGLRKDL
BZR-BAM_Agathis robusta            140 LSIDNSLELMEEEQVQEFIPKEPERDFAGTPYVPVYVMLPLGIINDRCQLVDPEGLRKDL
BZR-BAM_Podocarpus rubens          140 MSIDNSLELMEEE--QDFFPKEPERDFAGTPYVPVYVMIPLGIINDRCQLVDPEGLQKDL
BZR-BAM_Cupressus dupreziana       141 MSIEDSLELMEDERVQEFLPKEPERDFAGTAFVPVYVMLPLGIINDRCQLVDPECLRKDL
BZR-BAM_Taiwania cryptomerides     154 MSIENPLELMEDEHVQEFLPKEPERDFAGTPFVPVYVMLPLGIINDHCQLVDPEGLRKDL
BZR-BAM_Stangeria eriopus          139 VSVDNSLELFKDEQVQEFFPKEPERDFAGTSYIPLYVMLPLGILNDRCQLVDPEGLRKDL
BZR-BAM_Encephalartos barteri      130 ---DNSLELFKDEQVQEFLPKEPERDFAGTSYIPLYVMLPLGILNDRCQLVDPEGLRKDL
BZR-BAM_Ophioglossum vulgatum      144 RMPGKNSSITLVDQVRHGLISSHDNDFAGTPCIPVYVMIPLSFFNDKYQVTDTGMLRDYL
```

```
BAM2_Arabidopsis thaliana          123 RTLKSVNVDGVMVDCWWGIVESHTPQVYNWSGYKKLFQMIRELGLKIQVVMSFHECGGNV
BAM7_Arabidopsis thaliana          276 RILKSIHVDGVKVDCWWGIVEGHSPQEYNWTGYRQLFQMVRDLNLKIQVLMSFHECGGNV
BAM7_Amborella trichopoda          278 RILKSINVDGVMVDCWWGIVESHSPLEYDWHGYKKLFQIIRELKLKLQVVMSFHECGGNV
BAM8_Amborella trichopoda          268 RRLKSINVDGVIVECWWGIVEGWSPQKYIWSGYRELFNIVREVKLKLQIVMAFHECRANG
BAM8_Arabidopsis thaliana          283 SYMKSLNVDGVVIDCWWGIVEGWNPQKYVWSGYRELFNLIRDFKLKLQVVMAFHEYGGNA
BZR-BAM_Abies lasiocarpa           213 RALKSINVDGIMVDCWWGVVEGRTPQKYVWSGYRELFNIVREAKLKLQVVMSFHECGGTA
BZR-BAM_Pseudolarix amabilis       199 RALKSINVDGIMVDCWWGVVEGRIPQKYVWSGYRELFNIVREAKLKLQVVMSFHECGGTA
BZR-BAM_Pseudotsuga wilsoniana     216 RALKSINVDGIMVDCWWGVVEGRTPQKYVWSGYRELFNIVREAKLKLQVVMSFHECGGTA
BZR-BAM_Picea engelmandii          215 RALKSINVDGIMVDCWWGVVEGRTPQKYVWSGYRELFNIVREAKLKLQVVMSFHECGGTA
BZR-BAM_Tsuga heterophylla         216 RALKSINVDGIMVDCWWGVVEGRTPQKYIWSGYRELFNIVREAKLKLQVVMSFHECGGTT
BZR-BAM_Wollemia nobilis           203 RTLKSINVDGIRVDCWWGVVEGRTSQKYVWSGYRDLFNIVREAKLKLQVAMSFHEYGGNV
BZR-BAM_Agathis robusta            200 RTLKSINVDGIRVDCWWGVVEGRTSQKYVWSG----------------------------
BZR-BAM_Podocarpus rubens          198 RTLKSINVDGIMVACWWGIVESRTPQKYVWSGYRELFNIVRDAKLKLQVAMSFHECRGN-
BZR-BAM_Cupressus dupreziana       201 RTLRSINVDGIMVSCWWGIVEGRIPQKYVWSGYRELFDIVQEAKLKLQVSMSFHECGGNV
BZR-BAM_Taiwania cryptomerides     214 KTLRSINVDGIMVNCWWGIVEGRTPQKYVWSGYRELFDIVREAKLKLQVAMSFHECGRNV
BZR-BAM_Stangeria eriopus          199 RILKSVNVDGIMVDCWWGLVEGRTPQKYVWSGYRELFNIVRETKLKLQVVMSFHECVGNV
BZR-BAM_Encephalartos barteri      187 RTLKSVNVDGIMVDCWWGLVEGRTPQKYIWSGYRELFNIVRETKLKLQVVMSFHEYFD--
BZR-BAM_Ophioglossum vulgatum      204 KALKAAAVDGVTVECCWGLIERSIPQQYDWSGYEQLFGLIKEADLKLQVSMSFHEYIDG-
```

```
BAM2_Arabidopsis thaliana            183 GDDVHIQIPEWVREIGQSNPDIYFTDSAGRRNTECLTWGIDKQRVLRGRTALEVYFDYMR
BAM7_Arabidopsis thaliana            336 GDDVCIPLPHWVAEIGRTNPDIYFTDREGRRNPECLSWGIDKERILRGRTALEVYFDYMR
BAM7_Amborella trichopoda            338 GDDVCIPLPHWVVEIGKSNPDIFFTDREGRRNPECLTWGIDKERVLRGRTALEVYFDFMR
BAM8_Amborella trichopoda            328 GSEMSIPLPKWILEIGKDHQDIYFTDRERRRNVECLSWGIDKQRVLKGRTAIEVYFDFMR
BAM8_Arabidopsis thaliana            343 SGNVMISLPQWVLKIGKDNPDIFFTDREGRRSFECLNWSIDKERVLHGRTGIEVYFDFMR
BZR-BAM_Abies lasiocarpa             273 GDEVNIPLPKWILEIGKDNPDIYFTDREGRRNTECLTWGIDKERVLKGRTAVEVYFDYMR
BZR-BAM_Pseudolarix amabilis         259 GDD---------------------------------------------------------
BZR-BAM_Pseudotsuga wilsoniana       276 GDDVNIPL----------NPDIFFTDREGRRNTECLTWGIDKERVLKGRTAIEVYFDYMR
BZR-BAM_Picea engelmandii            275 GDDVNIPLPKWILEIGKDNPDIFFTDREGRRNTECLTWGIDKERVLKGRTAVEVYFDYMR
BZR-BAM_Tsuga heterophylla           276 GDNVNIPLPKWILEIGKDNPDIYFTDREGRRNTECLTWGVDKERVLKGRTAVEVYFDYMR
BZR-BAM_Wollemia nobilis             263 GDEVHIPLPKWILEIGKDNPNIFFTDREGRRNTECLTWGIDKERVLKGRTAVEVYFDYMR
BZR-BAM_Agathis robusta                  ------------------------------------------------------------
BZR-BAM_Podocarpus rubens            257 GDDVHIPLPKWILEIGKDNPDIFFTDREGRRNTECLTWGIDKERILKGRTAVEVYFDYMR
BZR-BAM_Cupressus dupreziana         261 RDEVHIPLPRWVLEIGRENADIFFTDREGRHNTDCLTWGIDKERVLKGRTALEVYFDYMR
BZR-BAM_Taiwania cryptomerides       274 GDEVHIPLPRWVLEIGRENTDIFFTDREGRRNTECLTWGIDKERVLKGRTALEVYFDYMR
BZR-BAM_Stangeria eriopus            259 GNDVNITLPKWILDIGRDNPDIFFTDREGRRNTECLT-----------------------
BZR-BAM_Encephalartos barteri            ------------------------------------------------------------
BZR-BAM_Ophioglossum vulgatum        263 --DVIITLPTWALEIGKENPDIYFTDQHGRRHSGCLTWGVDKERVFKGRNALEVYYDFMR

BAM2_Arabidopsis thaliana            243 SFRVEFDEFFEEKIIPEIEVGLGPCGELRYPSYPAQFGWKYPGIGEFQCYDKYLMNSLKE
BAM7_Arabidopsis thaliana            396 SFRIELAEFLEDGVISMVEIGLGPCGELRYPSCPIKHGWRYPGVGEFQCYDKYLSKSLRK
BAM7_Amborella trichopoda            398 SFRVEFDEFFADGVISEIEIGLGPCGELRYPSYPVRHGWRYPGIGEFQCYDQYLLKSLRK
BAM8_Amborella trichopoda            388 SFRMEFDDLFVESLISAVEIGLGPSGELQYPSFPERMGWRYPGIGEFQCYDKYLQHSLRK
BAM8_Arabidopsis thaliana            403 SFRSEFDDLFVEGLITAVEIGLGASGELKYPSFPERMGWIYPGIGEFQCYDKYSQLSLQK
BZR-BAM_Abies lasiocarpa             333 SFRMEFDDHFVDGAISEIEIGLGASGELKYPSYPERHGWRFPGIGEFQCYDKYMQKSLRK
BZR-BAM_Pseudolarix amabilis             ------------------------------------------------------------
BZR-BAM_Pseudotsuga wilsoniana       326 SFRMEFDDLFVDGAISEIEIGLGASGELKYPSYPERHGWRYPGIGEFQCFDKYMQKSLRK
BZR-BAM_Picea engelmandii            335 SFRMEFDDLFMDGAISGIEIGLGASGELKYPSYPERHGWRYPGIGEFQCYDKYMQKSLRK
BZR-BAM_Tsuga heterophylla           336 SFRMEFDDLFVDGAISEIEIGLGASGELKYPSYPERHGWKYPGIGEFQCYDKYMQKSLRK
BZR-BAM_Wollemia nobilis             323 SFRTEFDDLFIDGAISQIEIGVGASGELRYPSYLERHGWKYPGIGEFQCYDKYLQKSLRK
BZR-BAM_Agathis robusta                  ------------------------------------------------------------
BZR-BAM_Podocarpus rubens            317 SFRMEFDDLFVDGVISEIEIGLGHFGELRYPSYPERHGWTYPGIGEFQCYDKYLQKSLRK
BZR-BAM_Cupressus dupreziana         321 SFRMEFDDLFVDGAISVIEIGLGASGELRYPSYPERNGWKYPGIGEFQCYDKYLQKSLRK
BZR-BAM_Taiwania cryptomerides       334 SFRMEFDDLFVDGAISEIEIGLGASGELRYPSYPESHGWKYPGIGEFQCYDKYLQKSLRK
BZR-BAM_Stangeria eriopus                ------------------------------------------------------------
BZR-BAM_Encephalartos barteri            ------------------------------------------------------------
BZR-BAM_Ophioglossum vulgatum        321 SFRQNFHNLFEDNTIVCVEIGLGAQGDLQYPSHARKHGWKYPGIGEFQCYDKYLQKSLKA

BAM2_Arabidopsis thaliana            303 AAEVRGHSFWGRGPDNTETYNSTPHGTGFFRDGGDYDSYYGRFFLNWYSRVLIDHGDRVL
BAM7_Arabidopsis thaliana            456 AAESRGHLFWARGPDNTGSYNSQPQGTGFFCDGGDYDGLYGRFFLKWYSQVLIDHADQIL
BAM7_Amborella trichopoda            458 AAETRGHFIWARGPDNAGCYNSRPHDTSFFCDGGDYDSYYGRFFLGWYSQVLIDHADRVL
BAM8_Amborella trichopoda            448 AAKLRGHSFWARGPDNAGQYNSQPHETGFFCDRGDYDSYYGRFFLQWYSQVLIDHADHVL
BAM8_Arabidopsis thaliana            463 EAKSRGFTFWGKGPENAGQYSSHPHETVFFQERGEYDSYYGRFFLNWYSQLLIGHAENVL
BZR-BAM_Abies lasiocarpa             393 AAEARGHSFWARGPDNAGYYNSQPHDTGFFRDGGDYNSYYGRFFLRWYSQGLIEHGDRVL
BZR-BAM_Pseudolarix amabilis             ------------------------------------------------------------
BZR-BAM_Pseudotsuga wilsoniana       386 AAEARGHSFWARGPDNAGYYNSQPHDTGFFRDGGDYNSYYGRFFLRWYSQGLIEHGDRVL
BZR-BAM_Picea engelmandii            395 AAEARGHSFWARGPDNAGYYNSQPHDTGFFRDGGDYNSYYGRFFLRWYSQGLIEHGDRVL
BZR-BAM_Tsuga heterophylla           396 AAEARGHSFWARGPDNAGYYNSQPYDTGFFRDGGDYNSYYGRFFLRWYSQGLIEHGDRVL
BZR-BAM_Wollemia nobilis             383 AAEARGHSFWARGPDNAGYYNSQPQETGFFCDGGDYDSYYGRFFLRWYSQVLLEHCDRVL
BZR-BAM_Agathis robusta                  ------------------------------------------------------------
BZR-BAM_Podocarpus rubens            377 AAEARGHSFWARGPDNAGYYNSQPQDTGFFCNGGDYDSYYGRFFLRWYSQVLIEHCDRVL
BZR-BAM_Cupressus dupreziana         381 AAEARGHSFWARGPDNAGFYNSQPRDTDFFCDGGDYDSYYGRFFLHWYSQVLIEHCDRVL
BZR-BAM_Taiwania cryptomerides       394 AAEARGHSFWARGPDNAGFYNSQPHGTDFFCDGGDYDSYYGRFFLRWYSQVLIEHCDRVL
BZR-BAM_Stangeria eriopus                ------------------------------------------------------------
BZR-BAM_Encephalartos barteri            ------------------------------------------------------------
BZR-BAM_Ophioglossum vulgatum        381 AAVARGHPEWGIGPLNTGHYNLCPQDIRFFCDGGDYDSFFGRFFLGWYSEVLIDH-----
```

```
BAM2_Arabidopsis thaliana        363 AMANLAFEGTCIAAKLSGIHWWYKTASHAAELTAGFYNSSNRDGYGPIAAMFKKHDAALN
BAM7_Arabidopsis thaliana        516 CLAKLVFDSSCIAAKLPDVHWWYRTASHAAELTAGFYNPSNRDGYSAIASTLKKHGATLS
BAM7_Amborella trichopoda        518 ALANLAFEGTRIAAKISGIHWWYKTASHAAELAAGFYNPCNRDGYSPIAQMLKKHEVALN
BAM8_Amborella trichopoda        508 ALANLAFEGTPIVAKIPAVYWWYRTASHAAELTAGFYNPSNQEGYTLILNILKKHSATFR
BAM8_Arabidopsis thaliana        523 SLANLAFEETKIIVKIPAIYWSYKTASHAAELTAGYYNPSNRDGYSLVFETLKKYSVTVK
BZR-BAM_Abies lasiocarpa         453 DLANLAFEGTRIAVKVSGIHWWYKTASHAAELTAGFYNSSNLDGYGPICQIVKKHNATFN
BZR-BAM_Pseudolarix amabilis         ------------------------------------------------------------
BZR-BAM_Pseudotsuga wilsoniana   446 GLANLAFEGTRIAVKVSGIHWWYKTASHAAELTAGFYNSSNLDGYGPICQIMKKHNATFN
BZR-BAM_Picea engelmandii        455 DLANLAFEGTRIAVKVSGIHWWYKTASHAAELTAGFYNSSNLDGYGPVCQIMKKHNATFN
BZR-BAM_Tsuga heterophylla       456 DLANLAFEGTRIAVKVSGIHWWYKTASHAAELTAGFYNSSNLDGYGPICQIVKKHNATFN
BZR-BAM_Wollemia nobilis         443 TLANLAFEGTPIAVKVSGIHWWYKTASHAAELVAGFYNSSNMDGYAPICQVLKKHDATLN
BZR-BAM_Agathis robusta              ------------------------------------------------------------
BZR-BAM_Podocarpus rubens        437 SLANLAFEGTQIAVKLSGIHWWYKTSSHAAELAAGFYNSSNMDGYAPVCQVLKKYNATLN
BZR-BAM_Cupressus dupreziana     441 AFANFAFEGTQIAVKV--------------------------------------------
BZR-BAM_Taiwania cryptomerides   454 AFANLAFEGTQIAVKVSGIHWWYKTASHAAELAAGFYNSSNMDGYAPICQVLKKHDATFN
BZR-BAM_Stangeria eriopus            ------------------------------------------------------------
BZR-BAM_Encephalartos barteri        ------------------------------------------------------------
BZR-BAM_Ophioglossum vulgatum        ------------------------------------------------------------

BAM2_Arabidopsis thaliana        423 FTCVELRTLD--QHEDFPEALADPEGLVWQVLNAAWDASIPVASENALPCYDREGYNKIL
BAM7_Arabidopsis thaliana        576 FVSGEVQVLN--RPDDFSGALGEPEAVAWQVLNAAWDSGTPVARENSLACHDRVGYNKML
BAM7_Amborella trichopoda        578 FTCAELRTLD--QHEDFPEALADPEGLVWQVLNAAWDVGIPVASENALPCYDREGYNKVL
BAM8_Amborella trichopoda        568 FIFSGLQLTP----QDNDETMADPEGLTWQVLNAAWDQKMNIASQNAVPCYDREGFNRIL
BAM8_Arabidopsis thaliana        583 FVCPGPQMSP----NAHEEALADPEGLSWQVINAAWDKGLQIGGENAITCFDRDGCMRLI
BZR-BAM_Abies lasiocarpa         513 FSCVELNNLD--HEQGFLEAMEDPHGLVWQVMNAAWDAGIPVASVNPVPCHDRDGYNRIL
BZR-BAM_Pseudolarix amabilis         ------------------------------------------------------------
BZR-BAM_Pseudotsuga wilsoniana   506 FSCVDLNNLD--HEQGFLEAMEDPHGLVWQVMNAAWDAGIPVASVNPVPCNDRDGYNRIL
BZR-BAM_Picea engelmandii        515 FSCVDLNSLD--HEQGFLEAMEDPHGLVWQVMNAAWDAGIPVASVNPVPCYDRDGYNRIL
BZR-BAM_Tsuga heterophylla       516 FSCVDLNDLDHHHEQGFLEAMEDPHGLVWQVINAAWDAGIPVASVNPVPCHDRDGYNRIL
BZR-BAM_Wollemia nobilis         503 FTCVELHNLG--QEEGFLEAMEDSQGLLWQVLNAAWDARISVASINALPCYDRETYNRIL
BZR-BAM_Agathis robusta              ------------------------------------------------------------
BZR-BAM_Podocarpus rubens        497 FTCLEWHNLG---HEGFLEAMEESHGLTWQVLNAAWDSGIPVASTNTLPCYDRDSYNRIL
BZR-BAM_Cupressus dupreziana         ------------------------------------------------------------
BZR-BAM_Taiwania cryptomerides   514 FTCVEFPNLD--RDERLLEAMEDSQGLVWQVLNASWDAGIHVASVNALPCYDRDSYNRIL
BZR-BAM_Stangeria eriopus            ------------------------------------------------------------
BZR-BAM_Encephalartos barteri        ------------------------------------------------------------
BZR-BAM_Ophioglossum vulgatum        ------------------------------------------------------------

BAM2_Arabidopsis thaliana        481 ENAKPLTDPDGRHLSCFTYLRLNPTLMESQ-NFKEFE
BAM7_Arabidopsis thaliana        634 ESVKFRNDPDRKHLSSFAYSRLVPALMEGH-NIVEFE
BAM7_Amborella trichopoda        636 ENAKPAHDPDGRHLSAFTYLRLSPALMERQ-NFLEFE
BAM8_Amborella trichopoda        624 ETAKPRNDPDRHHLSSFAYQRMSPSLIQRASFFSEFD
BAM8_Arabidopsis thaliana        639 DIAKPRNHPDGYHFSFFTYRQPSP-LVQGSTCFPDLD
BZR-BAM_Abies lasiocarpa         571 ENAKPRNDPDGRHLIAFTYLRLSPTLMERE-NFCEFD
BZR-BAM_Pseudolarix amabilis         -------------------------------------
BZR-BAM_Pseudotsuga wilsoniana   564 ENAKPRNDPDGRHLIAFTYLRLSPTLMEKA-NFCEFD
BZR-BAM_Picea engelmandii        573 ENAKPRNDPDGRHLIAFTYLRLSPTLMERA-NFCEFD
BZR-BAM_Tsuga heterophylla       576 ENAKPRNDPDRRHLIAFTYLRLSPTLMERA-NFCEFD
BZR-BAM_Wollemia nobilis         561 ENAKPRNDPDRRHLVTFTYIRLSPTLMERT-YFSEFD
BZR-BAM_Agathis robusta              -------------------------------------
BZR-BAM_Podocarpus rubens        554 ENAKPMDDPDRRHLVTFTYVRLSPTLMERT-YFCEFD
BZR-BAM_Cupressus dupreziana         -------------------------------------
BZR-BAM_Taiwania cryptomerides   572 ENAKPRNDPDRRHLVAFTYLRLSPTLMERT-YFYEFD
BZR-BAM_Stangeria eriopus            -------------------------------------
BZR-BAM_Encephalartos barteri        -------------------------------------
BZR-BAM_Ophioglossum vulgatum        -------------------------------------
```

```
BAM2_Arabidopsis thaliana       518 RFLKRMHGEAVPDLGLAPGTQETNPE
BAM7_Arabidopsis thaliana       671 RFVKKLHGEAVMNHHHHHHQQV---
BAM7_Amborella trichopoda       673 RFVKRMHGEAVPDPQL---------
BAM8_Amborella trichopoda       661 RFVKSMHGEAVIDAQV---------
BAM8_Arabidopsis thaliana       639 YFIKRMHGD-IRDKQF---------
BZR-BAM_Abies lasiocarpa        676 RFVKRMHGEAVLNFHV---------
BZR-BAM_Pseudolarix amabilis        -------------------------
BZR-BAM_Pseudotsuga wilsoniana  601 RFVKRMHGEAVLNLHV---------
BZR-BAM_Picea engelmandii       610 RFVKRMHGEAVLNFHV---------
BZR-BAM_Tsuga heterophylla      613 RFVKRMHGEAVLNLHV---------
BZR-BAM_Wollemia nobilis        598 RFLKRMHGEAVLDLQ----------
BZR-BAM_Agathis robusta             -------------------------
BZR-BAM_Podocarpus rubens       591 RFVKRMHGEAVLDLR----------
BZR-BAM_Cupressus dupreziana        -------------------------
BZR-BAM_Taiwania cryptomerides  609 RFVKRMHGEAVLHFQ----------
BZR-BAM_Stangeria eriopus           -------------------------
BZR-BAM_Encephalartos barteri       -------------------------
BZR-BAM_Ophioglossum vulgatum       -------------------------
```

Additional File 7: Protein alignment of 13 BZR-BAM fusion proteins of conifers (highlighted in blue), cycads (yellow) and ferns (green). BZR-BAMs of *Arabidopsis thaliana* and *Amborella trichopoda* have been included for comparison, as well as the closest Arabidopsis ortholog lacking a BZR domain (BAM2). The position of the BZR1-like domain as well as the start of the β-amylase domain are indicated in the alignment.
